# A new scenario of hypothalamic organization: rationale of new hypotheses introduced in the updated prosomeric model

**DOI:** 10.3389/fnana.2015.00027

**Published:** 2015-03-19

**Authors:** Luis Puelles, John L. R. Rubenstein

**Affiliations:** ^1^Department of Human Anatomy, School of Medicine, University Murcia and Instituto Murciano de Investigación BiosanitariaMurcia, Spain; ^2^Nina Ireland Laboratory of Developmental Neurobiology, Department of Psychiatry, University of California, San FranciscoSan Francisco, CA, USA

**Keywords:** peduncular hypothalamus, terminal hypothalamus, acroterminal domain, genoarchitecture, anteroposterior pattern, dorsoventral pattern, length axis, tracts

## Abstract

In this essay, we aim to explore in depth the new concept of the hypothalamus that was presented in the updated prosomeric model (Puelles et al., 2012b; Allen Developing Mouse Brain Atlas). Initial sections deal with the antecedents of prosomeric ideas represented by the extensive literature centered on the alternative columnar model of Herrick (1910), Kuhlenbeck (1973) and Swanson (1992, 2003); a detailed critique explores why the columnar model is not helpful in the search for causal developmental explanations. In contrast, the emerging prosomeric scenario visibly includes many possibilities to propose causal explanations of hypothalamic structure relative to both anteroposterior and dorsoventral patterning mechanisms, and insures the possibility to compare hypothalamic histogenesis with that of more caudal parts of the brain. Next the four major changes introduced in the organization of the hypothalamus on occasion of the updated model are presented, and our rationale for these changes is explored in detail. It is hoped that this example of morphological theoretical analysis may be useful for readers interested in brain models, or in understanding why models may need to change in the quest for higher consistency.

## Introduction

**Figure 1:**
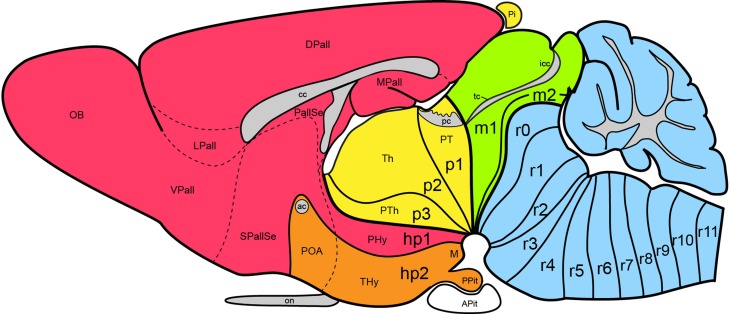
**Updated prosomeric model as applied to the adult mouse brain**. Hindbrain rhombomeres and cryptorhombomeres (r0–r11) are in blue, midbrain mesomeres (m1–m2) in green, diencephalic prosomeres (p1–p3) in yellow, and hypothalamo-telencephalic prosomeres (hp1–hp2) in red and orange, respectively. The roof, alar, basal and floor parts are not differentiated, for simplicity, but exist in every case (note the anterior commissure represents the rostralmost roof domain; the rostralmost floor corresponds to the mamillary area-M). Abbreviations: ac, anterior commissure; cc, corpus callosum; VPall, LPall, DPall, MPall, ventral, lateral, dorsal and medial pallial sectors; PallSe, pallial septum; SPallSe, subpallial septum; OB, olfactory bulb; POA, preoptic area; THy, terminal hypothalamus; PHy, peduncular hypothalamus; PTh, prethalamus; Th, thalamus; PT, pretectum; M, mamillary body; APit, anterior pituitary; PPit, posterior pituitary; pc, posterior commissure; tc, tectal commissure; icc, intercollicular commissure.

The hypothalamus is a brain region whose name is familiar to all neurobiologists, though not many claim to understand perfectly its position, limits and inner structure in the context of surrounding forebrain territories. Indeed, there is controversy even among experts about the morphological model that best accounts for its complexity. How the hypothalamus is regionalized during development is still largely a matter of conjecture, despite various lines of insight, such as its ancestral origin in chordates, an ample number of neurogenetic and genoarchitectonic studies, and identification of various candidate patterning mechanisms. Our anatomic knowledge of the complex nuclear composition of the hypothalamus is still redolent of the frustrating “potatoes- in-a-potato-sack” approach, though modern genoarchitectonic analysis has introduced a measure of order and promises rational classification. As a consequence of the remarkable structural heterogeneity of the hypothalamus, the logic of its intrinsic circuitry at the service of various functional systems operating throughout the brain and beyond (e.g., neurohumoral functions) remains obscure. However, we do know that the hypothalamus is an important central station involved in networked neural control of organismic humoral homeostasis, circadian neural activity patterns, self-placing computation, motor control and central drives. We clearly need deeper understanding of the genetic causal mechanisms that organize primarily hypothalamic structure and function, prior to the intervention of postnatal epigenetic plasticity. This requires an appropriate morphological model, pregnant with suggestions about the spatial dimensions and limits of potential causal signaling effects, which can be tested experimentally. There is a recently updated version of the prosomeric model (Allen Developing Mouse Brain Atlas reference atlases and ontology; Martínez et al., 2012; Puelles et al., 2012b, 2013, 2014; Puelles, 2013) that includes novel anatomical hypotheses about hypothalamic organization (Figure 1). These hypotheses possibly need an explanatory commentary, and this is the aim of the present essay.

## Antecedents of the updated prosomeric model

**Figure 2:**
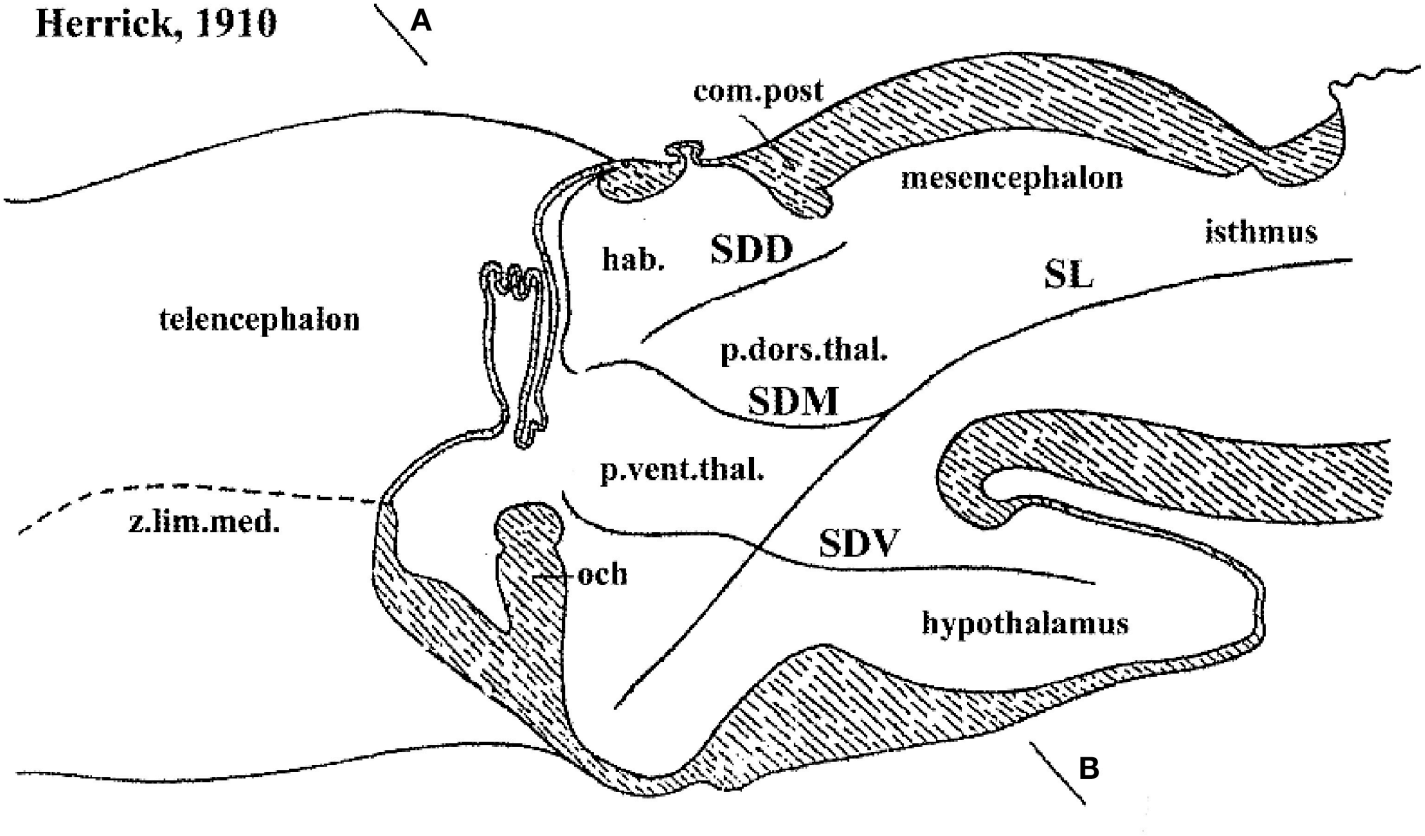
**Columnar model schema of Herrick (1910), depicting the forebrain of an urodele amphibian, here flipped horizontally and with redrawn lettering**. Note Herrick still used in this figure the then standard axial forebrain landmark—the sulcus limitans of His (SL)—, and represented relative to it the dorsal (SDD), middle (SDM) and ventral (SDV) diencephalic sulci. SDM and SDV clearly intersect the SL nearly orthogonally, though described in the text as “longitudinal.” The plane AB was presented as a diencephalic “cross-section.” In ulterior publications of Herrick the SL was no longer represented and the new columnar axis parallel to the SDM/SDV sulci and continuing to a telencephalic end was implicitly established, without ever having been defined by its creator.

**Figure 3:**
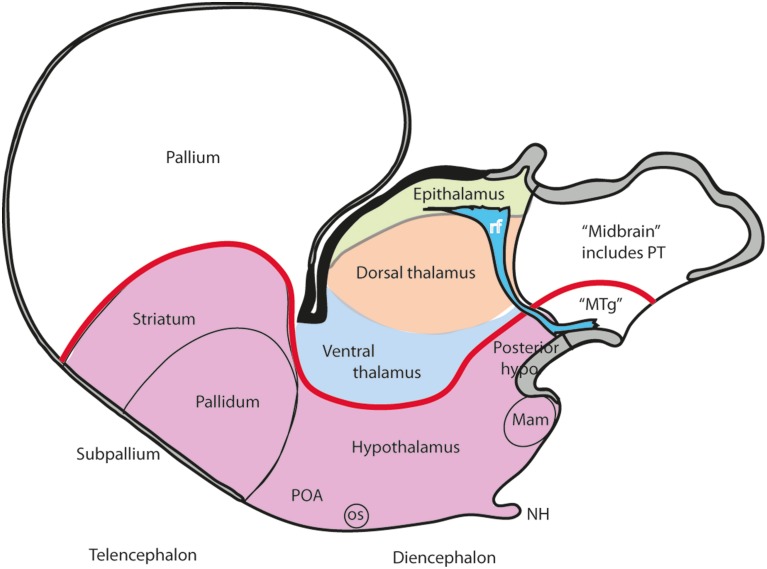
**Schema illustrating the modern columnar model of Swanson (1992, 2003), in which the essential features of the Herrick schema are conserved, while the hypothalamus is defined explicitly as the diencephalic basal plate (note this requires that the alar ventral thalamus is continuous with the telencephalic pallium, a point negated by fate and gene mappings)**. In this model all the thalamic zones and the posterior hypothalamus contact the midbrain. This is achieved by arbitrary inclusion of the pretectum in the midbrain and ascription of the diencephalic tegmentum to the posterior hypothalamus (this places a large part of the diencephalic substantia nigra inside the “hypothalamus”).

Hypothalamic studies during the last 100 years were largely interpreted using the columnar morphological model, which holds that the hypothalamus is the ventralmost longitudinal column of the diencephalon, and is intercalated between the telencephalon rostrally and the midbrain caudally. This concept was introduced by Herrick (1910) in amphibians (Figure 2), and was elaborated by Kuhlenbeck (1927, 1973) and others (e.g., Swanson, 1992, 2003; Alvarez-Bolado and Swanson, 1996) for vertebrates in general (Figure 3). We hold that this model is incorrect as applied to the forebrain, since its fundamental underpinning holds that the length axis of the neural tube ends beyond the diencephalon in the telencephalon (a position that we regard as arbitrary, and devoid of developmental correlation with axial mesodermal structures). In the original Herrick model the hypothetized columnar sectors of the forebrain neural wall were delimited by ventricular sulci (Figure 2); in general, such landmarks do not coincide with the boundaries of gene expression discovered in recent times (see Figure 3 in Puelles and Rubenstein, 1993). Molecular boundaries are thought to be much stronger and comparatively conserved limits, since they reflect primary causal regionalization features; the differential molecular identities of the limited territories control all subsequent histogenesis such as proliferation, neurogenesis and mantle development. On the other hand, ventricular sulci form as tertiary epiphenomena of mantle development; they emerge later, between the variously bulging parts of the differentiating mantle layer. It also was held in columnar theory that the resulting forebrain subdivisions—epithalamus, dorsal thalamus, ventral thalamus, hypothalamus—are associated with sensorimotor viscero-somatic functions analogous to those of brainstem columns; this tenet has aged considerably in the meantime. Importantly, the columnar model offers no account about the developmental mechanisms that might generate the postulated organization, nor explains causally finer regionalization within the columns (e.g., nuclear subregions). The recent loss of favor of this model has been accelerated by its apparent inability to integrate meaningfully the accruing variety of gene expression patterns observed in the developing diencephalon and hypothalamus (Puelles et al., 2004, 2012b; Shimogori et al., 2010; Diez-Roux et al., 2011).

A modified version of the columnar model is favored by Swanson (1987, 1992, 1993, 2003, 2012), and Alvarez-Bolado and Swanson (1996), who adapted to it the alar/basal concepts of His (1893a,b, 1895, 1904), extrapolating them from the diencephalon into the telencephalon, while maintaining the standard columnar axis of Herrick/Kuhlenbeck. In this model the telencephalic subpallium is held to be “basal,” and the pallium “alar,” while the hypothalamus represents the “basal” part of the diencephalon (Figure 3). However, there is no clearcut molecular evidence or causal underpinning that supports these changed notions of His' alar and basal plates (His, 1889, 1892, 1893a,b, 1894, 1895, 1904), which apparently simply answer to the preconceived idea of the axis ending in the telencephalon. Swanson and colleagues have performed extensive connectivity studies addressing the rat hypothalamus in the context of major forebrain functional circuits, significantly advancing functional analysis of the observed systems (Swanson, 1987, 2000a,b, 2003, 2007, 2012; Petrovich and Swanson, 2001; Sawchenko et al., 2000; Thompson and Swanson, 2003, 2010). We hold that all conclusions on these topics can be reinterpreted without significant loss using our non-columnar morphological model—the prosomeric model (Figure 1). Other recent authors who developed their own variant version of a columnar model with an axis ending in the telencephalon are Altman and Bayer (1986, 1988, 1995). These authors contributed extensive reports on developmental neurogenetic patterns in the rat hypothalamus. Part of their data and conclusions also can be translated into prosomeric terms, though some of their interpretations on diencephalic regionalization (e.g., the extent of the thalamic progenitor domain—in which both the prethalamus and the pretectum, and some aspects of hypothalamic partition, are misrepresented) seem inconsistent with recent gene expression studies and the phenotypes of some mouse mutants.

The abundance of molecular, genetic and developmental data now provides opportunity to investigate more fully the developmental organization of the hypothalamus. The title of this essay—“A new scenario of hypothalamic regionalization”—refers to the prosomeric model approach, which emphasizes a return to the length axis originally defined by His (1889, 1892, 1893a, 1894, 1895, 1904), and a detailed morphologic and molecular analysis of the hypothalamus along redefined dorsoventral and anteroposterior dimensions (Figure 1). This novel approach appears capable of improving experimental developmental analysis and, therefore, causal understanding.

## Comparison of the explanatory capacity of columnar and prosomeric (neuromeric) models

**Figure 4:**
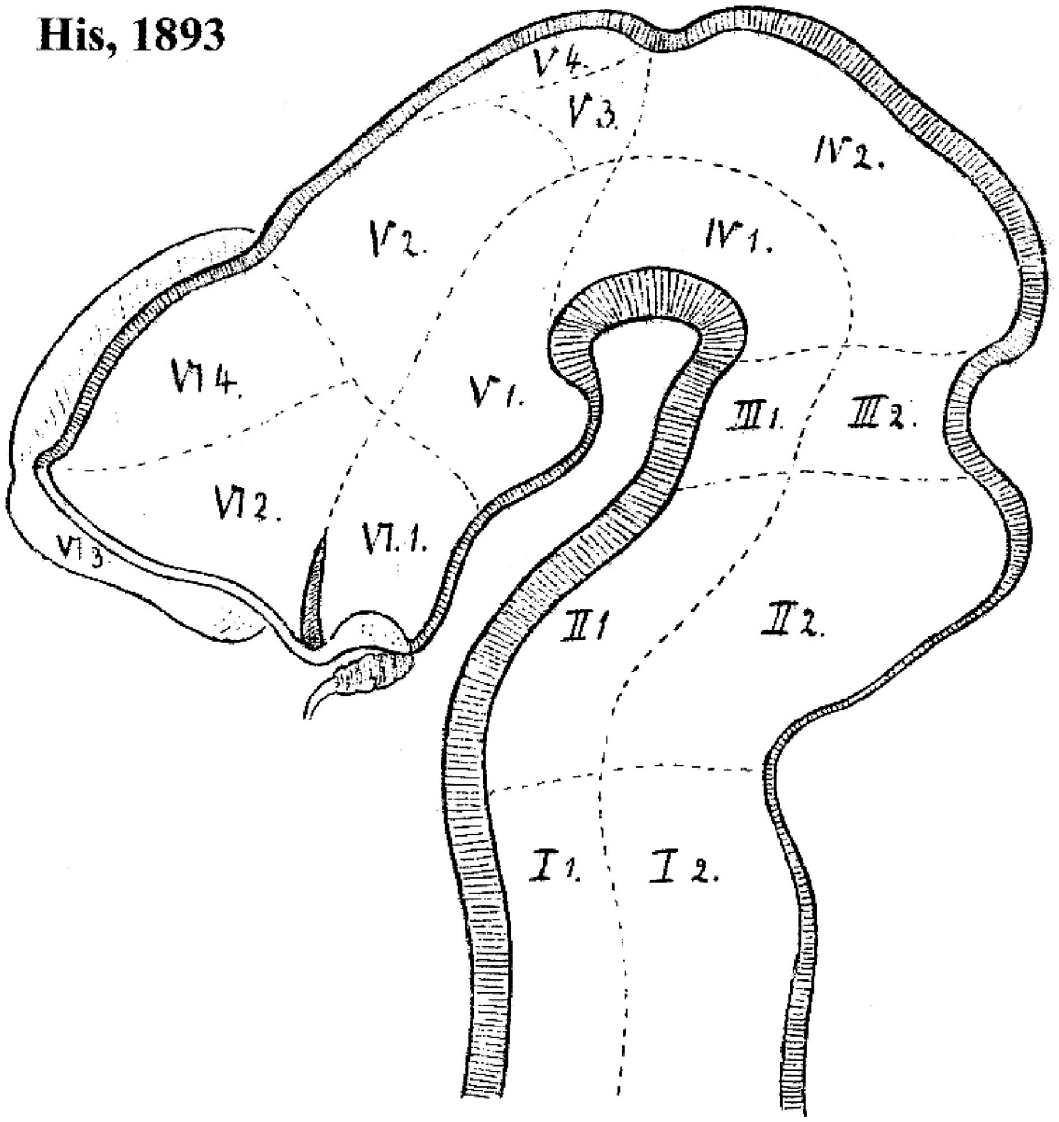
**Forebrain subdivision model of His (1893b)**. Note the overall course of the sulcus limitans, which in principle represents the alar-basal boundary. The hypothalamus of His is limited to his VI.1 and V.1 regions (optic and mamillary parts of the hypothalamus, respectively). Note the optic part (this is actually the tuberal basal part plus a part of the alar plate, including the suprachiasmatic primordium, as we understand the area now) is continuous dorsally with the telencephalic regions VI.2 (supposed to be subpallium, but including alar hypothalamus) and VI.4 (pallium); VI.3 is the olfactory bulb. On the other hand, the region V.1 (mamillary pouch) is held to relate dorsally with V.2, the thalamus. V.3 and V.4 possibly represent the pretectum and epithalamus, respectively. Note the oblique rostral border of the midbrain, which His (1893b) explained explicitly as an “arbitrary provisional line,” due to the lack of data about its proper placement at that time.

**Figure 5:**
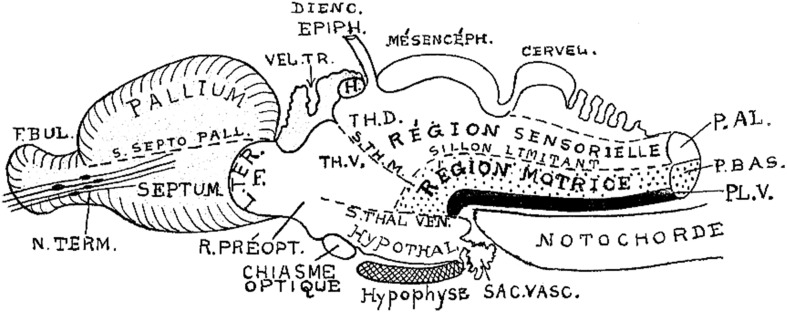
**Brain subdivisions in a generalized vertebrate model as conceived by Kappers (1947)**. Note conservation of the sulcus limitans of His (“sillon limitant”), and the clear concept of the sensory and motor longitudinal brainstem zones plus the floor plate (P.AL; P.BAS; PL.V). These bend uniformly around the cephalic flexure and end at the rostrally placed hypothalamus. The middle and ventral thalamic sulci (S.TH.M; S.TH.VEN) are represented as strictly transversal relative to the longitudinal dimension, which obviously does not end in the telencephalon, but in the hypothalamus. Note the notochordal tip contacting the mamillary pouch.

It usually is not recognized that the columnar model, perhaps because of its selective functional orientation, was not a helpful morphological framework toward understanding the mechanisms underlying forebrain development. Notably, this paradigm admitted over the years numerous inconsistencies and points of *impasse* (see examples below). We believe its assumptions have represented an occult obstacle to progress, mainly due to the pragmatic introduction by Herrick (1910) of an *arbitrary*, non-causally underpinned axis concept, which can be tracked in the literature of the next 100 years as a subconsciously implemented dogma that never was criticized or corrected by his followers. Indeed, when Herrick (1910, 1933, 1948) and Kuhlenbeck (1973) highlighted the idea of diencephalic columns, they unwittingly discarded the causal explanatory advantages of the earlier axial structural concept of His (1893a,b, 1895, 1904). This author had underlined the *epichordal* longitudinal position of the histogenetically precocious forebrain basal plate, whose growth mechanically causes the emergence of the alar/basal sulcus limitans; this longitudinal basal zone was held to end rostrally at the tuberal suboptic hypothalamus (Figure 4). Wilhelm His, one of the pioneers of neuroembryology, also defined the telencephalon as a *dorsal outgrowth* of the rostral alar plate, while his nascent term “hypothalamus” was restricted to the underlying forebrain basal plate (His, loc. cit.; review in Puelles et al., 2012b). These early ideas implicitly supported the *transversal* nature and general comparable dorsoventral patterning of the mesencephalic, pretectal, thalamic, prethalamic, and hypothalamic parts of the alar plate, as was also understood by Kappers (1929; 1947; Figure 5). This pioneering forebrain axial concept was based on comparative neuroembryological analysis in various vertebrates (His, 1889, 1892), and significantly included a topographic correlation of the floor and basal plates with the underlying notochord (ulteriorly shown experimentally to be causal). In contrast, Herrick (1910) did not adopt his new axis ending in the telencephalon because of any developmental discoveries. He rather assumed this ending *against available developmental knowledge*, because it was a “convenient” measure (Herrick, 1948), in order to be able to *call* “longitudinal” diverse diencephalic and telencephalic regions separated by ventricular sulci, which otherwise would be transverse in the model of His (Figure 4; compare Kappers, 1929, 1947; Figure 5). In this case the interpretation clearly preceded the concept. Herrick was interested in exploring the potential of forebrain regions delimited by sulci as *longitudinal* histogenetic and functional entities (so that they could be described as *columns*, and be assigned columnar functions extrapolated from the hindbrain), and he accordingly postulated the axis that was convenient to that purpose. This was done implicitly, without any argumentation, simply by saying the diencephalic sulci were longitudinal. He never defined his forebrain axis expressly, or discussed in detail the reasons for his modification of the axis. The functions alluded by Herrick were represented by the then novel insights about sensorimotor viscerosomatic functional specialization of the hindbrain nuclear columns associated to cranial nerves. Herrick thought that these notions might illuminate as well the functions of the forebrain, if it was similarly composed of four longitudinal columns, like the hindbrain. The potential functional light thus brought into the forebrain was the apparent reason why the axial change was accepted by the scientific community, though probably with little understanding of the morphologic price paid (with exceptions, such as Kappers, loc. cit.). Neuroanatomists understandably were then not much preoccupied with a developmental understanding of how a given structure was organized, or evolved. The emphasis was on gaining functional understanding, and this was also the apparent reason why the pre-existent segmental/neuromeric morphological models of brain development and structure (reviewed in Von Kupffer, 1906; Ziehen, 1906) practically fell into disuse after 1910: no function was attached to the observed neuromeres, whereas the columns apparently were functional entities. Curiously, nobody ever articulated thoughts about why longitudinal ventricular sulci should correlate with functional regionalization. Nowadays, the rare authors still attached to sulci have not reacted yet to the fact that developmental genetic patterning does not correlate topographically with ventricular sulci, a point already emphasized by Puelles and Rubenstein (1993). Indeed, sulci do not seem to be genomically coded—why should they? In other words, why should Nature select for brain ventricular sulci?

**Figure 6:**
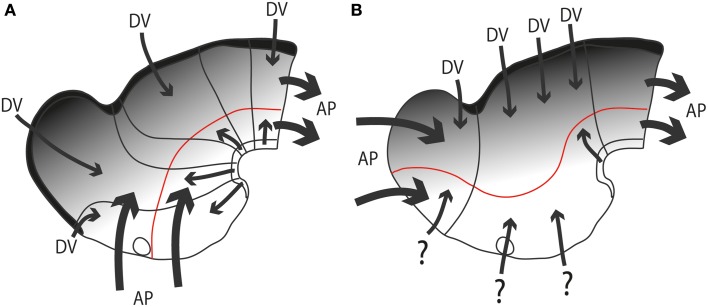
**Diagrams illustrating hypothetic anteroposterior patterning forces (AP, large thick arrows) and antagonistic dorsoventral patterning effects spreading from the roof and floor plates (DV, thinner arrows and gradiental shadowing) in the updated prosomeric model (A) vs. Swanson's columnar model (B)**. The postulated alar-basal boundary is marked in red in both cases. The postulated hypothalamic and diencephalic neuromeres are held to be patterned and delimited due to AP effects, as shown in **(A)**. In contrast, the columnar model implicitly holds that AP effects guide the division into telencephalon, diencephalon and midbrain **(B)**. The question marks of some arrows in **(B)** indicate the lack of notochordal and floor plate support for ventralizing effects at these sites (compare with **A**). The roof plate concept is also different in both models (thick black line).

As causal developmental neurobiology finally advanced in more recent times, a harmful effect of the now dogmatically accepted columnar axis was to change the expected source of anteroposterior patterning effects from the front of the hypothalamus (as implied by the neuromeric models, following His, 1893a,b) into the front of the telencephalon (as implied by columnar models) (Figures 6A,B). Secondly, the columnar idea of the hypothalamus as a ventral part of the diencephalon significantly handicapped the interpretation of dorsoventral patterning effects in this area as we understand them now, introducing much confusion. The columnar dorsoventral dimension of the diencephalon is not orthogonal to the essential axial landmarks, the forebrain floorplate and the notochord (Figure 6B).

The arbitrary columnar axis became an undoubted dogma after nearly a century of columnar thinking and publication. Most neuroscientists regard it as an established fact, rather than as a conjecture. Consequently, visualization of alternative interpretive causal possibilities was handicapped, and even the fact that this was happening was unnoticed among authors, reviewers and journal editors. This hidden effect that promotes wrong morphologic and causal assumptions may be easily traced in the relevant literature dealing with forebrain patterning, even up to contemporaneous reports; there is much inconsistent or non-substantiated axis-referred reasoning that distorts or misdirects causal analysis.

An example of such unnoticed explanatory inconsistency is the following: the columnar hypothalamus was postulated as the ventralmost diencephalic column, continuous with the telencephalic subpallium rostrally and the midbrain tegmentum caudally, though there is no notochord “under” the subpallium and the hypothalamus, as there is under the midbrain and hindbrain tegmentum (presently, the notochord represents the known causal agent of floor plate and basal plate induction, and resulting ventralization of the ventral part of the neural wall; Echelard et al., 1993; Roelink et al., 1994; Marti et al., 1995; Müller et al., 2000; Rastegar et al., 2002). The postulated rostral “basal” tissue is thus implied to arise on the whole out of dissimilar causal conditions (question marks in Figure 6B). Nobody apparently feels the need to explain this singularity. Another well-known example of inconsistency is that the columnar model does not explain the holoprosencephalic syndrome, in which abnormal rostral forebrain patterning causes the telencephalon and eyes to lose their bilateral division. Eventually, they can be completely lost, accompanied by the hypothalamus, leaving a stunted diencephalon remnant. Such a major patterning defect would be predicted to alter dorsoventral patterning of the whole diencephalon, since dorsalized structures normally equilibrate with ventralized counterparts, but the patterning of the prethalamus, thalamus and epithalamus appears normal in this syndrome. A final example of inconsistency: the basal plate is widely held to be “motor” in function, but the sensory eyes develop out of the hypothalamic region, as revealed by the position of the eye stalks and the arrival of the optic nerves at the optic chiasm, consistently with results from fate mapping the neural plate in several species (Rubenstein et al., 1998); therefore, the columnar viewpoint that the hypothalamus is the basal part of the diencephalon (Swanson, 1992; Figure 3) implicitly holds that the sensory eye is basal in origin, and we are forced to accept that the central optic pathway first enters and connects with the hypothalamus, the pretended diencephalic basal plate, before it reaches dorsally placed centers of analysis in the alar plate (note no other brain sensory input does this). No one in the ample columnar literature has ever discussed this point.

Columnar tradition has thus by action or omission caused scientific thought to stop at these and many other *impasse* points, since the columnar model and its cryptic axis only allows the thought “how odd,” but no further line of reasoning, causing unconscious evasive action (e.g., Swanson, 1992, 1993, 2003, 2012; see also Alvarez-Bolado and Swanson, 1996; their Figure 17). The cause of the problem is the use of Herrick's hundred-year-old incorrect longitudinal axis. Dozens of such inconsistent stumbling blocks can be pointed out, of which the scientific community does not seem to be aware, since attention to them is clearly not demanded by the peer review system.

Weighty molecular and experimental patterning evidence now shows that Herrick's diencephalic “columns” are not organized developmentally as dorsoventrally arranged structures, but as alar parts of *transverse neuromeric units*, or brain segments, which are themselves arranged rostrocaudally along the histogenetic axis defined by His (1893a,b) (Figure 6A). Each neuromere possesses its own sector of roof, alar, basal and floor longitudinal zones, a shared basic structural feature which makes all neuromeres fundamentally comparable developmental units, that is, *metameres*, irrespective of their mutual differences. We also know why this is so: all neuromeres share crucial dorsoventral patterning mechanisms that start at neural plate stages, which relate to comparable antagonistic floor plate and roof plate patterning signals throughout the axial dimension of the neural tube (Figure 6A; Puelles, 1995, 2013; Shimamura et al., 1995; Puelles and Rubenstein, 2003; Martínez et al., 2012). The floor plate is induced by the notochord (Echelard et al., 1993; Roelink et al., 1994; Marti et al., 1995; Müller et al., 2000; Rastegar et al., 2002; Sanchez-Arrones et al., 2009). This explanation does not apply in the columnar model for the diencephalon and telencephalon, since these pretended AP units do not share DV patterning mechanisms, either among themselves, or with the midbrain and hindbrain (Figure 6B).

**Figure 7:**
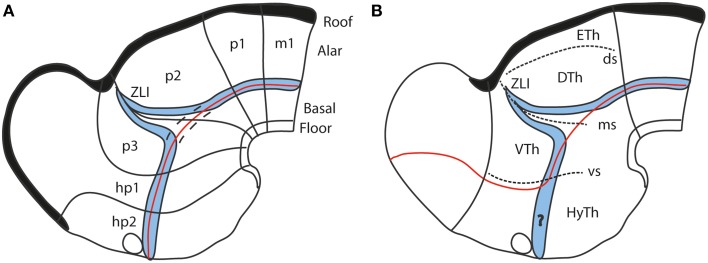
**Diagrams comparing how the domain of expression of *Nkx2.2* (in blue) relates to the alar-basal boundary (red line) in the updated prosomeric model (A) and the columnar model (B)**. The neuromeres are marked for reference in **(A)**, as well as the dorsal/middle/ventral diencephalic limiting sulci (ds, ms, vs) in **(B)**. Note the transverse ZLI spike of the *Nkx2.2* domain that separates thalamus and prethalamus is a secondary feature, due to the induction of this gene adjacent to the border of *Shh* expression, which is ectopically activated at the core of the ZLI. At neural plate and early neural tube stages, the expression band is strictly longitudinal (marked by dashes in **A**). In the columnar model **(B)**, the correspondence of the boundary with the gene band is disrupted at the arbitrary deviation of the former into the telencephalon. Moreover, note this model cannot explain why the gene band extends into the hypothalamus, cutting it into two halves, which cannot be understood as alar and basal parts of the hypothalamus, as in **(A)** (question mark in **B**).

It was repeatedly underlined (Puelles and Rubenstein, 1993; Shimamura et al., 1995; Puelles, 1995; Puelles et al., 2004, 2012b, 2014) that a thin longitudinal band expressing the transcription factor *Nkx2.2* courses through midbrain, diencephalon and hypothalamus along the apparent alar-basal boundary, or next to it; the topography of this band is comparable in all vertebrates investigated so far. This pattern emerges at neural plate stages, before the neural tube axis starts bending (Shimamura et al., 1995), and remains topologically invariant as the cephalic flexure forms (see Hauptmann et al., 2002). Secondarily, the band deforms around the transverse *Shh*-positive core of the zona limitans intrathalamica (ZLI; Figure 7). This very robust result (the neuronal derivatives of the band can be traced into the adult brain) reflects the common position of the alar-basal border throughout the forebrain and midbrain, and is clearly inconsistent with the columnar axis and the attached concept of diencephalic columnar subdivision. The *Nkx2.2* expression pattern obviously is *longitudinal* except at the ZLI, but does not enter the telencephalon (as predicted by Swanson, 2003, 2012). Moreover, it consistently divides serially the midbrain, diencephalon and hypothalamus into alar and basal moieties (Figure 7). An alar hypothalamus is impossible in the columnar model (thus the problem with the eyes). Nowadays we know that *Nkx2.2* expression is induced at high concentrations of the diffusing SHH morphogen at the border of basal plate (midbrain and forebrain) and floor plate (hindbrain and spinal cord) expression of *Shh*, which in its turn depends causally on notochordal signals (Echelard et al., 1993; Roelink et al., 1994; Marti et al., 1995; Rastegar et al., 2002). This result falsates the columnar postulate of the hypothalamus and telencephalic subpallium as an entirely basal region (Swanson, 1992, 2003, 2012).

Finally, the modern hypothalamus is not a homogeneous territory. Puelles et al. (2012b) mapped molecularly 33 discrete hypothalamic progenitor areas, and suggested that these areas produce a minimum of 150 derived nuclei or distinct cell populations. More recent data suggest that many hypothalamic areas are capable of sequentially producing several cell types over time; this extends significantly the list of different derivatives (Díaz et al., 2014). In contrast, columns were theoretically expected to be homogeneous (their components supposedly being unified by their dedication to a shared function; Kuhlenbeck, 1973). Obviously, this simplistic early columnar idea has evolved into the present-day concept of functional columnar subsystems of the hypothalamus, formed by strings of nuclei (Swanson, 2003, 2012), but remains devoid of any developmental explanation of how the hypothalamic column becomes subdivided into the 150 nuclei.

**Figure 8:**
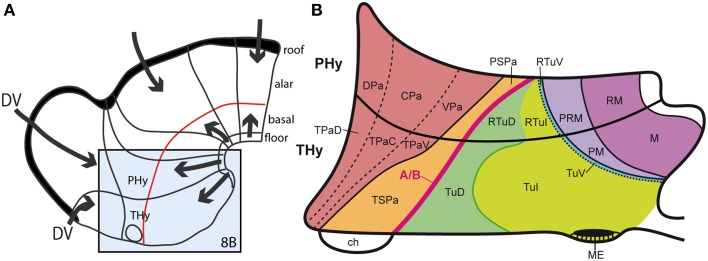
**(A)** Summary of antagonistic dorsoventral patterning effects spreading from the roof plate, including its rostralmost portion at the anterior commissure, and the floor plate, including its rostral hypothalamic sector. These effects presumably establish the alar-basal boundary (red line), as well as the telencephalo-hypothalamic boundary. The blue boxed area is examined in detail in **(B)**. **(B)** Map of the known dorsoventral molecular regionalization of the alar and basal hypothalamus, held to result from graded finer interactive effects within the primary dorsoventral pattern. The alar-basal boundary is marked by the thick red line. The alar longitudinal domains are represented by the paraventricular area (subdivided into dorsal, central, and ventral microzones) and the subparaventricular area (this relates to the optic chiasm and the initial course of the optic tract). The basal hypothalamus consists of similarly dorsoventrally related tuberal and mamillary regions (*sensu lato*). The updated terminology proposes distinguishing tuberal (Tu) from retrotuberal (RTu) areas, as well as perimamillary and mamillary *sensu stricto* (PM, M) from periretromamillary and retromamillary *sensu stricto* areas (PRM, RM), respectively belonging to THy and PHy. Note the Tu/RTu complex can also be subdivided dorsoventrally into dorsal, intermediate and ventral microzones (TuD, TuI, TuV; RTuD, RTuI, RTuV).

As mentioned, the nuclear structure of the hypothalamus is quite varied in terms of molecular profiles, neuronal aggregates and characteristic cell types (e.g., Swanson, 1987, 2003, 2012; Shimogori et al., 2010; Puelles et al., 2012b; Puelles, 2013). Its genoarchitectural profile, when interpreted within the updated prosomeric model, highlights a series of molecularly distinct parallel progenitor bands arranged along the dorsoventral dimension, that is, stacked one upon another between the dorsal hypothalamo-telencephalic boundary and the ventral hypothalamic floor plate (Morales-Delgado et al., 2011; Puelles et al., 2012b; Díaz et al., 2014; Domínguez et al., 2015; Santos-Durán et al., 2015). These progenitor bands clearly must result from the interplay of early antagonistic dorsalizing and ventralizing patterning mechanisms (Figures 8A,B). The corresponding developmental units underlie causally the well-differentiated adult paraventricular, subparaventricular, tuberal, perimamillary, and mamillary histogenetic areas, and the respective derived nuclear regions. Note the first two bands are alar, whereas the remaining three are basal, clearly emphasizing antecedent DV patterning, i.e., dorsalization vs. ventralization (Figure 8). These *dorsoventral* anatomic regions were always characterized instead as *anteroposterior* ones in columnar studies on the hypothalamus, in the absence of any postulates on corresponding AP patterning mechanisms that would cause such an organization (e.g., Swanson, 1987, 2003, 2012). We believe that the columnar viewpoint does not help causal explanation of this structural arrangement that typically subdivides the hypothalamus, because, surprisingly, no columnar assumptions whatsoever exist that predict and causally explain that columns eventually may show anteroposterior subdivisions (“how odd,” again). This problem is resolved by the neuromeres postulated in the prosomeric models, but these units are not admitted in columnar thinking. Examination of Figure 8B reveals that the dorsoventral alar and basal domains contemplated in the prosomeric model would require outrageously unparsimonious treatment in order to be interpreted as segmental units relative to the *columnar* axis. Again in this case the wrong axis impedes appropriate causal explanations to be conceived, and the issue is left unresolved.

Apart of the cited DV pattern, the updated prosomeric model contemplates also a general *anteroposterior* (AP) partition of the hypothalamus into *terminal* and *peduncular* transverse territories across the cited 5 DV bands (THy; PHy; Figure 8B). This partition implies the existence of an *intrahypothalamic* interneuromeric limit that separates the hypothalamo-telencephalic prosomeres 1 and 2 (hp1, hp2 in Figure 1; Pombal et al., 2009; Martínez et al., 2012; Puelles et al., 2012b, 2014; Puelles, 2013). These novel concepts take into consideration two classical telencephalic regions—the non-evaginated (impar) and evaginated (hemispheric) parts—that respectively complement the two hypothalamic AP regions at the dorsalmost part of the alar plate and the corresponding roof plate. This updated prosomeric pattern is rooted in the pioneering view of His (1893a,b), who already connected hypothalamus and telencephalon, seeing them jointly as a rostral forebrain unit; this feature changes significantly relative to earlier versions of the prosomeric model (**Figure 10B**: Puelles et al., 1987a, 2004, 2007; Puelles and Rubenstein, 1993, 2003; Rubenstein et al., 1994, 1998; Puelles, 1995, 2001, 2009). The hypothalamus + telencephalon forebrain unit (also known as secondary prosencephalon) is held to be the *rostralmost transversal part of the forebrain*, lying in front of the tri-neuromeric diencephalon proper (diencephalic prosomeres 1–3; p1–p3); the latter recovers in this model the original tegmental portions conceived by His (1893a,b, 1895), which had been arbitrarily ascribed to either hypothalamus or midbrain in the columnar tradition (e.g., compare Dong, 2008 with Puelles et al., 2012b; see also Figures 3 and **10B**).

**Figure 9:**
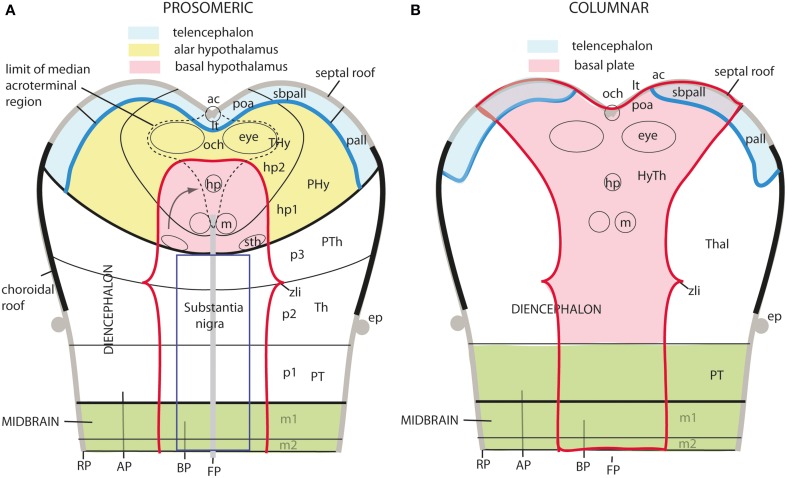
**Schematic comparison of the rostral end of the major longitudinal zones in flat neural plate maps, within the prosomeric model (A) and Swanson's columnar model (B)**. Structural landmarks which are conserved in both models are included to help fix the positions. In **(A)** both the basal and alar regions meet at the rostromedian terminal midline, intercalated between the end of the floor plate and the end of the roof plate (at the prospective anterior commissure–ac). The dashed lines delimit the acroterminal domain. Note the whole telencephalon (pallial and subpallial) relates ventrally with the hypothalamus and dorsally with the septal roof. In contrast, in **(B)** the hypothalamus is held to be continuous rostrally only with the telencephalic subpallium, but reaches itself the neural plate border, which is wrongly held to coincide with the optic chiasm and the lamina terminalis (because the preoptic area is ascribed to the hypothalamus). The telencephalic pallium is oddly depicted as being continuous caudally with the thalamus (the prethalamus, in fact); the comparison with **(A)** clearly suggests that a large part of the peduncular hypothalamus (PHy) is unwittingly ascribed to the “thalamus.” Another difference is observed in the rostral limit of the midbrain (green area).

The axial rostral neural tube sequence postulated in the prosomeric model accordingly runs: secondary prosencephalon-diencephalon-midbrain, each unit representing complete rings of the neural tube and of the forebrain (the hypothalamus/telencephalon composite is a modified ring, since it is closed rostrally by the *terminal wall* (Swanson, 1992). This singular morphologic feature can be visualized topologically via fate mapping at neural plate stages (Figure 9A). Its causal explanation relates to the fashion in which the floor plate and the roof plate end rostrally at neural plate stages (Shimamura et al., 1995; Puelles, 1995, 2013; Cobos et al., 2001; Sanchez-Arrones et al., 2009; Puelles et al., 2012b). Note that the only forebrain locus that fuses together during neurulation is the prospective roof plate (Cobos et al., 2001), and the telencephalon field present at neural plate stages is demonstrably parallel to this longitudinal zone (*loc. cit*.; Figure 9A). The telencephalon accordingly no longer can be explained as representing by itself the rostral end of the neural tube (compare Figure 9B). It is not more rostral than the eye and the whole hypothalamus; analogously to the eye, it should be considered a giant derivative that buds bilaterally out of the dorsal alar hypothalamus. Its patterning into subpallial and pallial moieties likewise starts at neural plate stages, this being a topologically AP pattern (the subpallium field lies rostral to the pallium field; Figure 9A); note columnar tradition has wrongly defined pallium/subpallium as a DV pattern, again causing unrecognized problems in causal interpretation (Figure 9B). The telencephalon accordingly is best understood as a hypothalamic derivative (also from the evolutionary point of view). Topologically, the hypothalamus is no hypo*thalamus*, but a hypo*telencephalon*.

## New aspects of the updated prosomeric model

The expression “new scenario” is used in the title because significant changes were introduced with regard to the preceding model version of Puelles and Rubenstein (2003) by Puelles et al. (2012b); these novelties also appeared in the Allen Developing Mouse Brain Atlas reference atlases and related ontology (www.developingmouse.brain-maps.com, online since 2009). Among the recent model changes are included various aspects that are not relevant for the hypothalamus, such as a better systematic treatment given to the telencephalic subpallium (see Puelles et al., 2013), a redefinition of pallial sectors and the concept of the claustrum (Puelles, 2014), and the introduction of the m2 mesomere and the cryptorhombomeres r7–r11 (Figure 1; Alonso et al., 2012; Puelles et al., 2012a; Tomás-Roca et al., 2015). We address here only the novelties that affect the hypothalamus, generally offering solutions for nagging conundrums that had resisted previous analysis. Our concern with some of these unresolved issues was expressed explicitly in the Puelles and Rubenstein (2003) review.

The old difficulties we now believe to have solved with the update are three: (1) the early topographic relationship of the hypothalamus with the notochord; our new analysis led us to molecular and causal redefinition of the hypothalamic *floor plate*, and we discovered its *epichordal character* throughout (important corollaries: there is no prechordal part of the neural tube, and the well-known median displacement of prechordal plate cells occurs *ventrodorsally* in front of the terminal hypothalamic wall); (2) we resolved satisfactorily the dorsalward course of the transverse intrahypothalamic boundary across the telencephalic field, in order to connect it with the roof plate (*impasse* on this in Puelles and Rubenstein, 2003); its ending at the floor plate was also modified; consequently, this limit acquires the topologic properties of a *complete neuromeric border* (see Puelles and Rubenstein, 2003) and the hypothalamus + telencephalon complex (the secondary prosencephalon) results divided in prosomeres hp1 and hp2; (3) the topologic position of the mamillary/retromamillary and tuberal regions in the basal hypothalamus was reconsidered, reaching the novel conclusion that both regions are *longitudinal*, rather than transversal (as we thought before); this led to the proposal of a novel partition, the *retrotuberal area*, as well as to the distinction of a similarly longitudinal intercalated domain between tuberal/retrotuberal and mamillary/retromamillary regions, the *perimamillary*/*periretromamillary area* [note we write “mamillary” with a single “m,” since we believe, following Rose (1939); Bleier (1961); Berman (1968), and (Jones, 1985), that the descriptor derives from the Latin term “mamilla” (nipple); otherwise we consequently should use “mammary” instead, if we held the descriptor derives from “mamma” (breast), but nobody does this].

A further significant change was applied to the updated concept of hypothalamus (Puelles et al., 2012b), attending to a difficulty that had not been noticed before, namely, (4) the need to explain the unique rostromedian hypothalamic specializations, a task achieved via the definition of the *acroterminal hypothalamic domain*.

## Rationales on these points

### Relationship of the hypothalamus with the notochord (hypothalamic floor plate)

**Figure 10:**
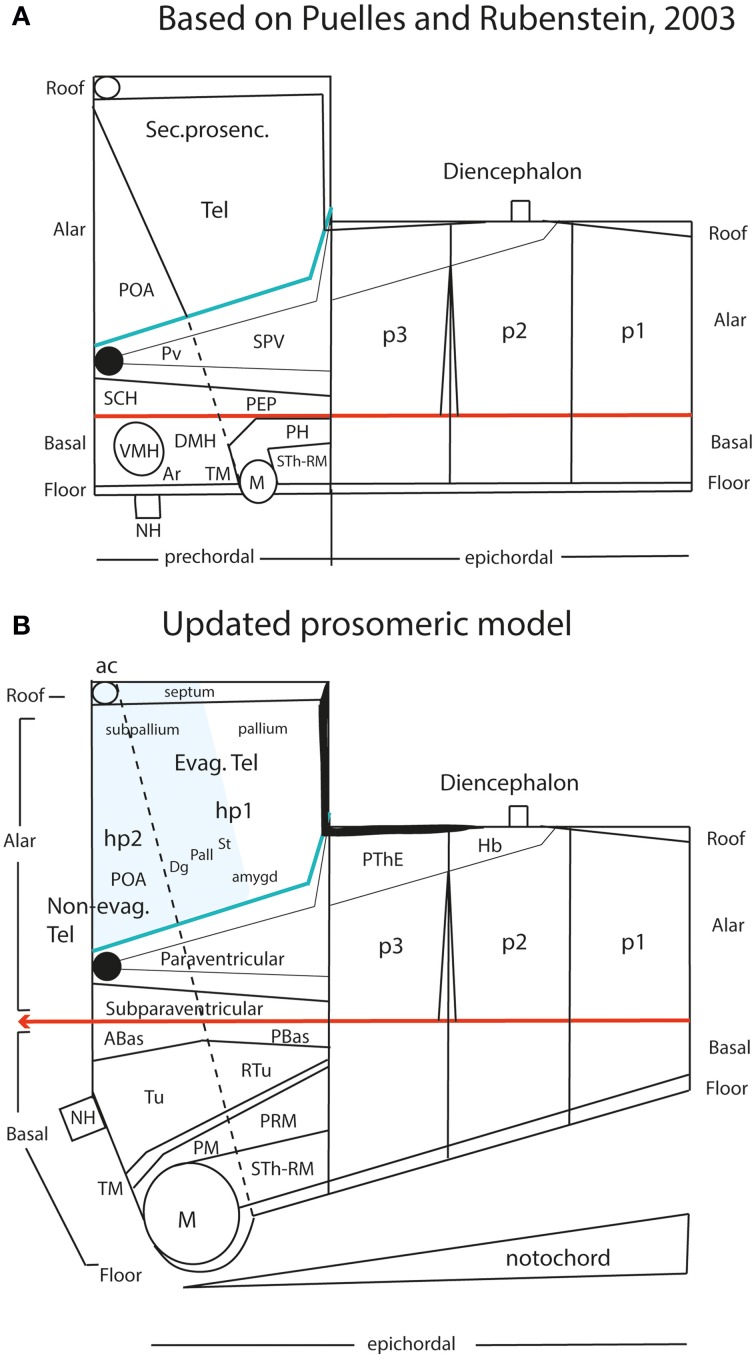
**Schematic comparison of the earlier prosomeric model version of Puelles and Rubenstein (2003) in (A) with the updated version of Puelles et al. (2012b) in (B)**. The **(A)** schema was slightly modified, repositioning more conveniently the anterior commissure, and eliminating for simplicity all unnecessary details in the present context. The **(B)** schema illustrates changes in the intrahypothalamic boundary, which now extends from the roof plate into the floor plate, distinctly separating the hp1 and hp2 prosomeres and the PHy and THy parts of the hypothalamus. The telencephalic subpallium is identified as a blue field; note its POA, Dg, Pal, and St parallel subdivisions. The alar hypothalamus remains essentially unchanged, apart the introduction of the paraventricular and subparaventricular areal names. The basal hypothalamus is deeply changed, due to our recognizing the mamillary area as occupying an extreme rostral and ventral longitudinal position, consistently with the new floor concept, and the tip of the notochord. This pushes the whole tuberal area, including the median eminence, infundibulum and neurohypophysis (NH), out of the hypothalamic floor (compare **A**) and into the rostral end of the basal plate. It represents now a fully longitudinal domain. The novel retrotuberal area (RTu) lies caudally to the tuberal area *sensu stricto* (Tu), and extends back to the prethalamic (p3) tegmentum, dorsally to the periretromamillary area (PRM). Rostral to PRM lies the perimamillary band (PM).

In earlier versions of the prosomeric model, including Puelles and Rubenstein (2003), we held that the diencephalon and midbrain were *epichordal* (i.e., their floor plate was causally influenced by the underlying notochord), while the secondary prosencephalon, represented ventrally by the hypothalamus, was a *prechordal* rostral part of the neural tube (i.e., its floor plate lacked notochordal influences, and related causally instead to the prechordal plate mesoderm; Figure 10A). The implied prechordal floor region included retromamillary, mamillary and tuberal (median eminence, infundibulum and neurohypophysis) neighborhoods (Figure 10A). The histologic and functional variety shown by these regions was bewildering and difficult to explain causally, since there was no known property of the postulated prechordal plate induction that would account for these different structural fates. This was definitely a “how odd” situation needing attention within the prosomeric model. A wider concern lay in considering potentially unsatisfactory a forebrain axis that was defined by two different axial causes, the notochord up to the diencephalon and the prechordal plate more rostrally, insofar as these mesodermal derivatives are themselves molecularly distinct cell populations, though sharing secretion of the SHH morphogen. In the background of this concern was the apparently hard result suggesting that the entire forebrain vesicle of *Amphioxus* is epichordal (Hatscheck, 1882; Von Kupffer, 1893; Lacalli, 1996; Nieuwenhuys, 1998).

Our understanding of this difficulty was unexpectedly illuminated by the experiments of García-Calero et al. (2008) on temporally-stepped extirpation of the prechordal plate in early chick embryos. It was found that complete deletion of the prechordal tissue immediately after its formation caused a loss of the differentiation of the basal plate throughout the expanded forebrain (secondary prosencephalon, diencephalon, and midbrain), in addition to holoprosencephaly and massive molecular dorsalization of remnant tissue. Selective deletion of the notochordal tip only caused loss of the floor plate. Prechordal plate deletions performed after increasing time intervals—thus allowing prechordal cells to act upon the neural primordium during the interval—“saved” progressively the basal plate fate in caudorostral order (e.g., first midbrain, then diencephalon, finally hypothalamus; we had a selective early marker of the mamillary anlage, which was the last basal locus to appear). Even later deletions saved the holoprosencephaly syndrome, and finally also the loss of *Shh* expression in the subpallium (these clearly are prechordal effects on the alar plate). The conclusion was reached that the prechordal plate, a migrating cell population derived from the node (Izpisúa-Belmonte et al., 1993), sequentially exerts diverse inductive effects as it relates topographically to a sequence of basal and alar neural domains. Initially it is needed for the specification of the basal plate rostral to the isthmus, probably acting in parallel to notochordal and floor plate signaling upon this domain, but it does not itself induce floorplate-like structures, a feature particularly noted in the terminal wall, where prechordal signals work without accompanying chordal effects. The ventrodorsal migration of prechordal cells along the median terminal wall allows them to have additional specific effects, first on the hypothalamic rostromedian basal plate (where the tuberal infundibulum and the retrochiasmatic anterobasal area emerge), and then on the rostromedian alar plate, leading to separation of the eyes (chiasmatic area) and of the telencephalic vesicles (terminal lamina), ending, finally, with the specification of the preoptic patch of *Shh* expression, important for subpallial regionalization. The association of prechordal inducing effects to the basal zone, first, and to the alar zone, afterwards, was consistent with the novel idea that, topologically, the movement of prechordal plate cells is not rostralward, as it appears to naïve inspection, but *dorsalward* relative to the terminal wall (progressing from the tip of the floor plate to the tip of the roof plate). On the other hand, the notochordal founder cells also emerge from the node, but represent non-motile cells which incorporate sequentially to the caudally elongating chordal primordium along the body midline (the axis) as the node recedes caudalwards; vertical chordal signaling is known to induce specifically the differentiation of the floor plate in the neural ectoderm, a phenomenon starting already at neural plate stages (Echelard et al., 1993; Roelink et al., 1994; Marti et al., 1995; Rastegar et al., 2002; Sanchez-Arrones et al., 2009). These results led us to the conviction that causal underpinning of both the forebrain length axis and the floor plate should be only ascribed to the notochord, and we classified any prechordal plate patterning effects as separate *terminal* non-axial DV patterning mechanisms produced by a motile signal source.

**Figure 11:**
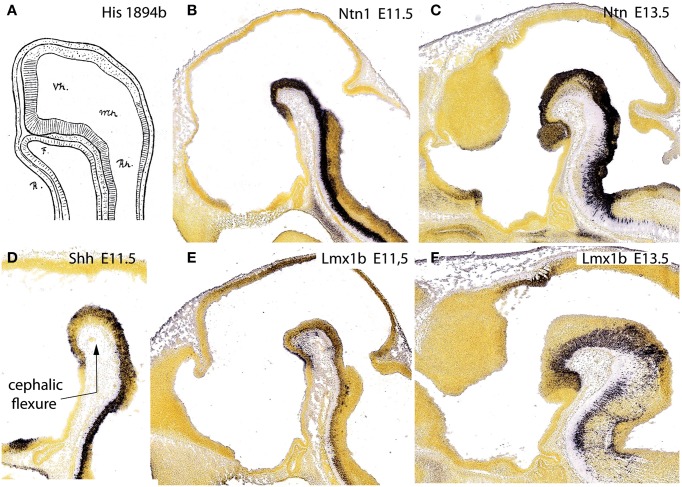
**Figure taken from Puelles et al. (2012b), illustrating in (A) the primordial intimate contact of the forebrain floor with the notochord, as well as the hypothalamic terminal plate closing rostrally the tube, from a drawing by His (1894) of a shark embryo**. **(B,D,E)** show the floor plate expression of three mouse genes, *Ntn1*, *Shh*, and *Lmx1b* at E11.5, displaying the same rostral end at the mamillary pouch; **(C,F)** show *Ntn1* and *Lmx1b* at E13.5, for clearer identification of the mamillary territory.

Our attention next turned to where lies precisely the rostral tip of the notochord relative to the hypothalamic primordium. We explored this issue in the literature, as well as via genoarchitectural analysis. We found that the literature is often vague and inconclusive about this point. Evidently, the notochord (or head process) only contacts the median floor of the neural primordium at very early stages (neural plate, early neural tube; see Figure 11A), since the morphogenetic appearance of the cephalic flexure soon causes the separation of these two tissues. Nevertheless, several credible images reported on such later stages show that the tip of the notochord usually contacts or approaches the mamillary pouch (e.g., Romanoff, 1960; Figures 85, 105, 335; diverse images in Kuhlenbeck, 1973; e.g., his Figure 48C). Consistently with this result, most workers describing the *earliest* topography of the notochord relative to the forebrain underlined a rostral end at or under the prospective mamillary pouch (e.g., His, 1893a,b; Von Kupffer, 1894, 1906; Jurand, 1974; Morris-Kay and Tuckett, 1987; Saucedo and Schoenwolf, 1994; Sulik et al., 1994; Puelles, 1995; Alvarez-Bolado and Swanson, 1996; Barteczko and Jacob, 2002; Bardet, 2007; Sanchez-Arrones et al., 2009). This agrees with observations of Johnston (1923) on the existence of a modified floor plate rostral to the isthmus, all the way to the mamillary area, a result which we reproduced with whole-mount histochemical labeling of an AChE-positive epichordal floor-plate strip ending at the mamillary area (Puelles et al., 1987a).

If we return to the provisional conclusion reached above that only the notochord induces a floor plate fate in the neural primordium (this can be correlated with incipient molecular differentiation of the floor plate already at open neural plate stages; Sanchez-Arrones et al., 2009), the literature data on the notochordal tip topography jointly point out that the forebrain floor plate must end beyond the prosomeric diencephalon, within the hypothalamus, and specifically at the mamillary pouch.

Moreover, we searched the Allen Developing Mouse Brain Atlas for floor-plate-specific gene markers, and found that not only *Shh* (which is directly induced in the floor by the notochord), but also *Foxa1*, *Lmx1b*, *Ntn1*, and *Nr4a2*, appeared expressed at the forebrain floor, with an identical rostral end. At E11.5, labeling ended rostrally at a small outpouching of the midline, which subsequently transformed into the mamillary pouch at E13.5 (Figures 11B–F). This genoarchitectural finding was revolutionary for both the columnar and earlier prosomeric models. In columnar models, the mamillary hypothalamic area is held to be a *caudal* diencephalic region (Figure 3), whereas the new results strongly support a position at the *rostral end* of the forebrain (encompassing the rostralmost floor). On the other hand, earlier versions of the prosomeric model (Figure 10A) had assumed that the forebrain floor reached the tuberal infundibular area, whereas the new results negated this possibility, suggesting that the tuberal region must be a rostromedian component of the basal plate (Figure 10B; see below).

Retrospectively, it may be noticed that the position of the mamillary area in the prosomeric model always was a difficulty. We had it initially in p4, caudal to p5 and p6 —Bulfone et al. (1993), Puelles and Rubenstein (1993)—, possibly due to the influence of His (1893a,b, 1895). Subsequently, we progressively felt the need to push it to a more rostral topologic position, as done in Puelles and Rubenstein (2003) (Figure 10A). Finally, we surprisingly found that it falls nicely at the absolute rostral end of the hypothalamic floor plate, coherently with various other novel morphologic features (Figure 10B; Allen Developing Mouse Brain Atlas; Puelles et al., 2012b). The resulting updated hypothalamic floor plate is thus shorter than previously imagined, but is entirely epichordal, thus parsimoniously unifying the causal underpinning of the forebrain axis throughout. Unexpectedly, our new interpretation also becomes consistent with the epichordal position of the entire brain in *Amphioxus*. Note we can now tentatively start to explain why other rostromedian territories in the hypothalamus (and beyond) differentiate distinctly than the floor plate, since they develop alternatively within the *median basal hypothalamus* (tuberal area), the *median alar hypothalamus* (chiasmatic area) or the *median preoptic telencephalon* (terminal lamina) (Figure 10B). All these non-floor median forebrain areas are sequentially influenced by prechordal signals in the absence of notochordal signals, and their distinctive structural and molecular profiles can be attributed confidently to DV patterning (not possible in the columnar model; Figures 6A,B; 8A). On the other hand, the true epichordal hypothalamic floor still shows two different regions—the mamillary and retromamillary floor domains—, an aspect which turns out to be consistent with our postulate of two hypothalamic prosomeres (Figure 10B; see below).

### Rostral end of the roof plate and full course of the intrahypothalamic boundary (=neuromeric border between hypothalamic prosomeres hp1 and hp2)

As reviewed in Shimamura et al. (1995) and Puelles (1995), the lateral border of the neural plate with the primitive non-neural ectoderm represents the prospective roof plate of the neural tube. The process by which the plate halves hinge upwards, and the bilateral borders then fuse together at the midline, forming the roof plate, is known as neurulation. The anterior and posterior neuropores are transiently open sites where the neurulation process has not yet finished. Puelles et al. (1987b) previously discussed the discrepant views in the literature about the closure of the anterior neuropore, bearing on the identification of the rostralmost roof plate point. They also performed a crucial experiment aimed to test the main hypotheses, by marking the rostromedian end of the anterior neuropore with a black plastic thread at successive stages in chick embryos. The results revealed that there is a single caudorostral sequence of closure of the anterior neuropore (other authors, as e.g., Swanson, 1992, still propose a double closure mechanism that so far lacks experimental support). It was suggested that the rostralmost roof plate roughly coincides with the prospective locus of the anterior commissure, that is, it would correspond to the telencephalon (earlier views had speculatively suggested several other possibilities apart this one, notably the optic chiasma; e.g., His, 1893a,b; Alvarez-Bolado and Swanson, 1996, their Figures 4, 16). Ulterior fate-mapping experiments on the median end of the roof plate were performed by Cobos et al. (2001) using quail-chick homotopic grafts; the results fully corroborated the earlier result of Puelles et al. (1987b), and distinctly identified the bed of the anterior commissure as the rostralmost locus of the forebrain roof plate. The crossing of the anterior commissure appears in all vertebrates at the upper end of the terminal lamina. It is simultaneously understood to represent the bottom end of the septal commissural plate, though it actually represents its *rostral* end, as indicated by these experimental data. These fate-mapping data about the morphologic signification of the median bed of the anterior commissure inescapably imply that the entire septal *midline* belongs to the forebrain *roof plate* (Figure 10B), contradicting the popular assumption that the septum is a “ventral component” of the subpallium (the paramedian septum containing bilaterally the major septal nuclei belongs instead to the telencephalic alar plate). The same fate-mapping data indicate that the preoptic terminal lamina is neither roof- nor floor-plate-derived, but a rostromedian *terminal alar* differentiation of the secondary prosencephalon, corresponding, jointly with the chiasmatic area, to the place where the right and left alar telencephalic fields are *primarily* continuous in the neural plate (i.e., there is no fusion here, since the continuity already exists in the open neural plate; Figures 9A, **13**).

Insofar as the prosomeric model postulates that the whole telencephalon is an alar derivative of the secondary prosencephalon that is topologically superposed dorsally to the alar hypothalamus, it poses no problem to realize that the roof plate corresponding to the hypothalamus is the telencephalic roof (Figures 10A,B). The same results lead to inconsistent and unparsimonious interpretations within the columnar model, wherein the basal plate is held to reach the septum (Figure 9B; Swanson, 1992).

Now, coming to our problem, if the hypothalamus is subdivided anteroposteriorly in two domains, as considerable morphologic evidence suggests (Puelles and Rubenstein, 2003; Puelles et al., 2012b), then the separating intrahypothalamic boundary might represent an interprosomeric limit. This is only possible, theoretically, in the case that this boundary was complete, that is, was traceable all the way from the floor plate into the roof plate (according to the criterion formulated by Puelles and Rubenstein, 2003). Therefore, it is not enough to show that the intrahypothalamic boundary divides the hypothalamus transversely; it needs to be shown that it also divides the overlying telencephalic field, and reaches the local roof plate. This is the point at which we stumbled with earlier versions of the prosomeric model, since we did not find a convincing solution for how this boundary might satisfy this criterion (several alternative options were considered in Bulfone et al., 1995; Shimamura et al., 1997; and Puelles, 2001; finally Puelles and Rubenstein, 2003 acknowledged an *impasse*; see Figure 10A). The reason of these failures turned out to be an error in our assumption of *where* was the bed of the anterior commissure in terms of telencephalic subpallial domains. Up to 2007 we had assumed that this median locus corresponded to the anterior entopeduncular area or AEP (now renamed *diagonal area* or Dg; see Allen Developing Mouse Brain Atlas, and Puelles et al., 2013; Figures 10A,B). This implied that the septal roof plate ended within the AEP/Dg, while the preoptic area was thought not to participate at all in the septal roof plate, lying wholly in the alar plate.

However, more precise genoarchitectural mappings (notably of the *Shh* expression pattern) performed in the mouse (Flames et al., 2007; Allen Developmental Mouse Brain Atlas) and the chick (Bardet, 2007; Puelles et al., 2007; García-López et al., 2008; Bardet et al., 2010; Medina and Abellán, 2012) eventually disclosed that the preoptic area shows dorsally a median spike-like region that reaches the rostralmost septal roof—the bed of the anterior commissure—in between the right and left diagonal domains (**Figure 13**). This allowed to relocate the anterior commissure, and, accordingly, the rostral end of the roof plate, to this dorsomedian preoptic region, which can be conveniently named *septo-commissural preoptic area* (SCPO; Allen Developmental Mouse Brain Atlas; note Medina and Abellán, 2012 identify this domain as “commissural preoptic area,” or POC, a term that in our opinion loses the semantic reference to a simultaneous ascription to the septum). The *median preoptic nucleus* that develops in the SCPO mantle zone is widely mapped in rodent atlases as surrounding frontally the anterior commissure in the median plane, consistently with this new interpretation (MnPO; **Figure 13**; see also Puelles et al., 2013).

**Figure 12:**
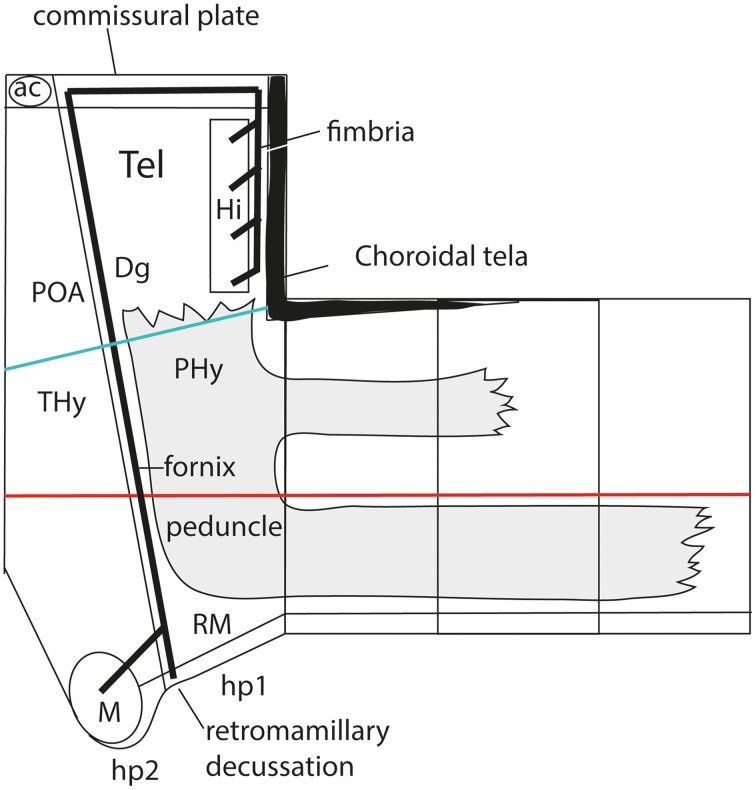
**Prosomeric interpretation of the course of the fornix and peduncular tracts within the updated model**. These two tracts are exclusively associated to the peduncular hypothalamus (PHy). The fimbrial fibers originate within the hippocampal complex, represented within the caudomedial pallium, next to the choroidal roof. They first course strictly longitudinally along the roof plate (septal commissural plate), but change course when they reach the hp1/hp2 boundary. Here they turn ventralwards entering a dorsoventral trans-hypothalamic route (via the rostral part of PHy) all the way into their final decussation within the retromamillary floor plate. Shortly before that, the fibers that innervate the mamillary body separate at right angles, and enter rostrally the basal hp2. The telencephalic peduncle (gray-colored) is first transverse while it courses dorsoventrally through the caudal part of the peduncular hypothalamus (next to the hypothalamo-diencephalic border); once it reaches the basal plate it bends backwards (knee around the subthalamic nucleus) and enters its descending longitudinal course through the diencephalic, midbrain and brainstem tegmentum. The upper root of the peduncle that carries thalamo-cortical and cortico-thalamic fibers through the alar prethalamus (reticular nucleus) is represented as well.

As a consequence, it soon became obvious that this conceptual change at the preopto-septal intersection allowed to extend the intrahypothalamic boundary into the roof plate according to a new possibility which had not been considered before, namely, following the boundary between the preoptic area and the diagonal area (the *preopto-diagonal border*; dash-line in Figure 10B). This solution of the old conundrum seemed satisfactory for various reasons. First, the boundary separates the non-evaginated preoptic area (the classic telencephalon *impar*) from the evaginated telencephalic vesicle; theoretically, this allows a tentative causal explanation of this morphogenetic difference as related to differential neuromeric molecular identities. Second, the preoptic area within hp2 is corroborated as a distinct telencephalic territory that relates intimately to the optic area (the evaginated eye vesicle and the optic chiasma), representing its immediate dorsal neighbor within the anterior part of the alar secondary prosencephalon, whereas the evaginated telencephalon within hp1, placed altogether *caudally* to the preoptic area, limits separately with the paraventricular hypothalamic alar area; this represents the frontier that is traversed selectively by the cerebral peduncle (Figure 12). Third, the well-known course of the fornix tract in front of the interventricular foramen, as it passes bilaterally behind the anterior commissure to enter the hypothalamus, suddenly acquired morphologic meaning, that is, the possibility of a causal explanation (there must be reasons for the course of any brain tract). Indeed, it can be hypothesized that, during their growth beyond the end of the hippocampal fimbria, the fornix tract fibers first elongate *longitudinally* along the paramedian septal commissural plate, that is, parallel to the roof plate; however, once they reach the preopto-diagonal boundary, most of them seem unable to cross it, and turn topologically 90° ventralward (forming the postcommissural fornix), to grow thereafter *dorsoventrally* along the caudal aspect of the intrahypothalamic boundary all the way to the retromamillary floor plate, where a number of the fornix fibers deccusate (Figure 12; see also Stanfield et al., 1987). Fourth, the new concept also apparently explains why the septal commissural plate consists of two different sectors, a caudal one containing the hippocampal and callosal commissures, and a rostral one containing the anterior commissure (Figures 10B, 12). Within the updated prosomeric model, the reason is that we deal here with the roof plate domains of two different neuromeres, hp1 and hp2, where distinct axonal navigational guidance mechanisms are expected. No previous explanation background existed before for the remarkable course of the fornix. Curiously, this background wholly disappears as soon as this solution for the completeness of the intrahypothalamic boundary is abandoned (returning to earlier prosomeric model versions, or to columnar models).

The hypothalamo/telencephalic roof plate (evolutionarily it was hypothalamic before it was telencephalic) is accordingly divided into preoptic and hemispheric sectors by the extended intrahypothalamic border, and, as mentioned above, mamillary and retromamillary sectors are distinguished at the hypothalamic floor plate. This boundary at the floor plate is likewise underlined by the behavior of the fornix tract, which seems to be guided dorsoventrally through the whole hypothalamus by the intrahypothalamic boundary (Bardet, 2007; Puelles et al., 2012b). The invariant dorsoventral course of the fornix tract ends with a crossing of the hypothalamic floor plate just caudally to the mamillary body, that is, in the retromamillary area (Edinger and Wallenberg, 1902; Ramón y Cajal, 1911; Valenstein and Nauta, 1959; Stanfield et al., 1987; Köhler, 1990). Note the latter is identified in columnar descriptions as the “supramamillary area,” though this traditional prefix is semantically inconsistent within the modified columnar schema used by Swanson (1987, 1992, 2003, 2012), in which the area is as retromamillary as in the prosomeric model, since it forms part of the same longitudinal zone, the postulated basal plate, topologically caudal to the mamillary body (Figures 3, 9). Interestingly, neurons of the retromamillary area project reciprocally to the dentate gyrus, which implies an inverse ventrodorsal route via the fornix (Pasquier and Reinoso-Suarez, 1978; Haglund et al., 1984; Nitsch and Leranth, 1994). Since the intrahypothalamic border lies just rostral to the fornix tract, it neatly separates the molecularly distinct mamillary and retromamillary areas, allowing these likewise to be explained as differential neuromeric AP phenomena (Figure 10B). An explanation of why these two neighboring regions are structurally and molecularly distinct had never been offered before. Of course, this interpretation of the fornix implies that the fornix fibers that target the mamillary nuclei (and several other cell populations, such as the ventromedial shell formation) must cross the intrahypothalamic border *rostralwards* to reach them (Figure 12; see in this respect Stanfield et al., 1987).

As a consequence of being able to define this transverse boundary all the way from the roof plate into the floor plate, using the fornix as a crucial anatomic landmark (apart other anatomic features summarized graphically by Díaz et al., 2014; their Figure 1), we postulated that the secondary prosencephalon (or hypothalamo-telencephalic complex) is divided into two prosomeres, identified as “hypothalamic prosomeres 1 and 2” (hp1, hp2). The numbering proceeds in caudo-rostral order, continuing the caudo-rostral sequence of the diencephalic prosomeres 1-3. We abstained purposefully from continuing the cardinal list of prosomeres—e.g., naming them p4 and p5—, since this surely would lead to confusion with our earlier (now obsolete) p1–p6 model (Bulfone et al., 1993; Puelles and Rubenstein, 1993), in which the hypothalamus was subdivided in three quite non-comparable prosomeres p4–p6, including a misconceived floor region.

We came up with the idea to call the hp1-hypothalamus “*peduncular hypothalamus*” (PHy), referring to its clearcut and constant relationship in all vertebrates with the dorsoventral hypothalamic course of the cerebral peduncle (Figure 12; note the observable basal bending of the peduncle caudalwards is not understood within the columnar conception, which holds the whole tract is longitudinal). The caudal boundary of the peduncle while it courses through the hypothalamus thus roughly marks the limit between the PHy and the diencephalic prethalamus (check the topology in Figures 9A, 10B, 12). The advantage of the non-topographic “peduncular” term is that it intentionally evades referring to the controversial axis, while alluding to a well-known landmark present in all vertebrates. Accordingly, it can be used by any neuroscientist, irrespective whether he/she believes the hypothalamic course of the peduncle is transverse (prosomeric model) or longitudinal (columnar model). For the hp2-hypothalamus we considered for a time the use of “prepeduncular” as descriptor, but discarded it because it would be more precise to say “prefornical,” since the fornix tract is the immediate peduncular landmark behind the intrahypothalamic frontier. Eventually, we chose to name this hypothalamic region “*terminal hypothalamus*” (THy; Allen Developing Mouse Brain Atlas; Puelles et al., 2012b, 2013; Puelles, 2013), in order to emphasize the relative position of this transverse unit at the topologic rostral end of the forebrain, leading to its implication in the “terminal wall.” The latter term was apparently introduced by Swanson (1992), aptly referring to the rostromedian region that closes rostrally the neural tube (Figure 11A; see below more details about this median locus).

THy is continuous dorsally with “its” telencephalic sector, the preoptic area (Figures 1, 10B); well-known terminal hypothalamic derivatives include in dorsoventral order the supraoptic, lateral anterior, suprachiasmatic, anterior, anterobasal, ventromedial, arcuate, and mamillary nuclei; there is also a terminal part of the dorsomedial nucleus, placed immediately caudal to the arcuate nucleus. Paradoxically, the terminal dorsomedial nucleus lies *ventral* to the ventromedial nucleus (this semantically confusing situation represents collateral damage of the columnar axis, to which all these classic terms refer; the new scenario demands complete revision and adjustment to the prosomeric “natural” axis of all positional descriptors in hypothalamic nomenclature).

On the other hand, PHy is continuous dorsally with the whole evaginated telencephalon (Figure 1), and includes as significant derivatives (again in dorsoventral order) the major part of the paraventricular nucleus, the peduncular part of the dorsomedial nucleus and the retromamillary area. Recently we have been searching the Allen Developing Mouse Brain Atlas for early gene expression patterns that are restricted to either the THy or the PHy, thus collectively defining molecularly the intrahypothalamic boundary. Part of these data are presented in this Issue by Ferran et al. (2015).

Interestingly, genoarchitectural data (Puelles et al., 2004, 2012b, 2014; Shimogori et al., 2010; Diez-Roux et al., 2011) show that PHy and THy are patterned dorsoventrally into a shared series of longitudinal zones across the respective alar and basal territories (Figure 8B). The alar-basal boundary is continuous with the diencephalic one (as was already recognized in the earliest versions of the prosomeric model; Figures 10A,B), and is marked by the dorsal boundary of the basal expression of *Shh* in the ventricular zone, which is partially overlapped by the above-mentioned longitudinal band expressing *Nkx2.2* (Figure 7). This molecular border, which roughly coincides with the sulcus limitans concept of His (1893a,b), reaches on both sides the terminal wall under the optic chiasm.

Leaving aside the alar telencephalic fields of hp1 and hp2, the subjacent alar hypothalamus shows a common longitudinal zonal division into a *paraventricular area* (Pa; we previously called it “supraopto-paraventricular area,” but later discovered that the supraoptic nucleus only appears within THy) and a *subparaventricular area* (SPa) (Figures 8B, 10B). The former is differentially labeled by *Otp* and *Sim1*, and lacks expression of *Dlx* or *Arx* genes, which are characteristic both of the overlying telencephalic subpallium and the underlying subparaventricular area. The peduncular paraventricular sector (PPa) is much broader than its companion terminal sector (TPa), and typically shows a tripartite triangular shape (DPa+CPa+VPa in Figure 8B). Its expands dorsoventrally caudalwards, toward the hypothalamo-diencephalic border, where it ends (it contacts there the prethalamic reticular nucleus and the overlying prethalamic eminence). PPa produces the largest part of the paraventricular nucleus complex, plus a radially migrated dorsal entopeduncular population. In contrast, the rather thin terminal paraventricular portion (TPa) relates to smaller parts of the paraventricular complex, namely the subpial supraoptic nucleus, the lateral anterior nucleus and the anterior periventricular area. Note the so-called “tuberal supraoptic nucleus,” which we prefer to call “tuberal suboptic nucleus,” according to its true position relative to the optic tract, lies in the underlying basal plate, though its neurons apparently migrate tangentially into this position from TPa origins (Morales-Delgado et al., 2011).

The underlying subparaventricular area differentially produces GABAergic neurons and also shows differently sized terminal and peduncular sectors (TSPa, PSPa; Figure 8B, 10B). In this case, TSPa produces more voluminous derivatives, including the suprachiasmatic nucleus and the main (classic) anterior hypothalamic nucleus. The PSPa component forms a posterior tail of the anterior hypothalamic nucleus, an area that can be also described topographically as a “preincertal area” (corresponding to the “subincertal area” of some rodent brain atlases), since it is continuous with the prethalamic zona incerta formation, with which the SPa shares various gene markers (Puelles et al., 2004, 2012b, 2014; Shimogori et al., 2010; Puelles, 2013).

The basal territories of hp1 and hp2 are very extensive dorsoventrally, compared with those of the rest of the forebrain, and, interestingly, basal THy is much larger than basal PHy (Figures 8B, 10B). This aspect may be due to early patterning influences of the prechordal plate, in concert with the predominant terminal expression of the early neural gene *Six3* (Lagutin et al., 2003). This basal domain was classically divided into tuberal and mamillary regions, traditionally interpreted as anteroposterior items within the columnar model. In all the prosomeric model versions advanced up to the Puelles and Rubenstein (2003) review (Figure 10A), we tentatively accepted an anteroposterior arrangement of these two regions within the hypothalamic basal plate, consistently with our misguided concept of the floor plate extent (see above). Nevertheless, there was dim awareness of unresolved problems there. Eventually a satisfactory solution was found for this aspect, which accordingly was changed in the Allen Developing Mouse Brain Atlas, as well as in Martínez et al. (2012), Puelles et al. (2012b, 2013, 2014), and Puelles (2013), as is explained in the next section.

### The topologic position of the mamillary/retromamillary and tuberal regions in the basal hypothalamus

The background for the search of a better solution for the hypothalamic basal pattern was represented in the first place by our noticing of the fact that some *longitudinal* lines extending rostralwards from the cephalic flexure seem to end by sweeping neatly around the mamillary region to meet the terminal wall (then supposed to be the floor plate). This implied an inconsistency (“how odd” situation), since a longitudinal line in the lateral wall should not meet the floor plate, being topologically parallel to it. For instance, Kuhlenbeck (1973) always traced the sulcus limitans of His into such a perimamillary ending; this feature of his thinking led him to define the tuberal hypothalamus as an alar plate derivative (a point recently taken again by Diez-Roux et al. (2011) on the basis of genoarchitectural considerations). A similar curve is also traced by the longitudinal course of the mamillotegmental tract. Vertebrate species showing a clearcut hypothalamic ventricular organ (a linear circumventricular specialization which is unremarkable in mammals; see review in Puelles et al., 2012b) likewise provide evidence suggesting that this organ curves longitudinally around the mamillary region. The dorsal premamillary nucleus bends similarly around the mamillary body, and so does the tuberomamillary population of histaminergic neurons. The conundrum to resolve obviously was that the mamillary region cannot be a longitudinal domain and simultaneously display a transversal border with the tuberal region (Figure 10A). One of these aspects must be illusory, and both required attention.

Our previous conclusion that the hypothalamic floor plate ends precisely at the mamillary area (see above) was significant in this regard, since the floor plate is a primary longitudinal reference. This result by itself weighs importantly in favor of considering the mamillary/retromamillary region a longitudinal zone, consistently with the course parallel to the floor of the mamillotegmental tract and the band of perimamillary grisea. *Dlx* and *Isl1* gene expression within the tuberal region distinctly limits the negative mamillary region along a curve that parallels the local floor plate (see Puelles et al., 2012b, their Figures 8–10). The same longitudinal boundary is underlined from the other side by genes selectively expressed within the mamillary and/or retromamillary areas, such as *Otp* and *Foxb1* (*ibid*). *Otp* expression highlights a curved tissue band within the mamillary region *sensu lato* that limits with the *Dlx/Isl1*-positive tuberal region. This is the band that produces the dorsal perimamillary nucleus within its terminal portion, and it was identified as the “*perimamillary/periretromamillary area*” (PM/PRM; Figures 8B, 10B; Simeone et al., 1994; Puelles et al., 2012b; Puelles, 2013; Allen Developing Mouse Brain Atlas; note the implied two parts correspond to THy and PHy, respectively). Close examination of these relationships suggested that the tuberal region *sensu lato*, which is quite massive rostrally (THy), extends longitudinally all the way to the hypothalamo-diencephalic boundary (PHy) via a gradually diminishing caudal portion placed over the PM/PRM; this “caudal tuberal” region in principle separates the mamillary region from the overlying alar-basal boundary (Figures 8B, 10B). This observation made it possible to regard the tuberomamillary boundary as purely longitudinal.

The same as the mamillary region *sensu lato* decomposes dorsoventrally into the dorsal PM/PRM and the ventral mamillary/retromamillary (M/RM) areas *sensu stricto*, the tuberal region *sensu lato* also can be subdivided dorsoventrally into three longitudinal subdomains, identified by Puelles et al. (2012b) as dorsal, intermediate and ventral, across both PHy and THy (Figure 8B). The dorsal subdomain encompasses the precociously differentiating cells of the classic hypothalamic cell cord, aggregated into the anterobasal and posterobasal areas (ABas, PBas; Figure 10B). The intermediate subdomain includes as its own derivatives the dorsomedial nucleus (which has peduncular and terminal parts) and the arcuate nucleus (also terminal), and receives as a migrated entity the ventromedial nucleus, which is produced at the dorsal subdomain (see Puelles et al., 2012b on this previously unknown feature). Finally, the ventral (or tuberomamillary) subdomain is rather thin and corresponds to the hypothalamic ventricular organ, being likewise the restricted source of histaminergic neurons (which partly invade neighboring mamillary areas (see Puelles et al., 2012b for data supporting this new point). It limits ventrally with the PM/PRM areas.

This analysis implies that the hypothalamic basal plate is patterned dorsoventrally into 5 longitudinal zones, all of which expand rostralwards in a fan-shaped configuration into their respective ends at the terminal wall (Figure 8B). The large intermediate tuberal subdomain significantly encompasses rostrally the median eminence, infundibulum and neurohypophysis. This solution of the hypothalamic basal plate problem is clearly satisfactory in that it allows to understand the whole alar and basal (plus telencephalic) patterning of the rostral forebrain as a special case of standard dorsoventral patterning, implying antagonistic dorsalizing and ventralizing signals diffusing from the roof and floor plates (Figures 6A; 8A), as occurs elsewhere in the neural tube (notably in the diencephalon and midbrain, where various relevant DV gene patterns are shared). The columnar model forbids such an explanation, due to its unhelpful axis reaching the telencephalon (Figures 6A,B), and does not provide a parsimonious alternative explanation.

We also reflected that the name *tuberal area* (Tu) strictly was meant originally only for the terminal (THy) sectors of these tuberal subdomains, since this term refers to the external bulge of the median eminence and infundibulum. The caudal, molecularly-defined “tuberal” extension into the peduncular (PHy) territory hardly relates to these rostromedian specializations, as it relates instead to the overlying peduncle. We therefore distinguished the caudal part of this basal complex with a novel term, the *retrotuberal area* (RTu), in analogy to the retromamillary neighbor (Figures 8B, 10B). Thus, within basal PHy we have the RTu and RM, with their respective five dorsoventral subdivisions (RTuD, RTuI, RTuV, PRM, RM), and within basal THy there appear the Tu and M regions, with their own five dorsoventral subdivisions (TuD, TuI, TuV, PM, M). See Shimogori et al. (2010), Puelles et al. (2012b) and Ferran et al. (2015) for details of differential gene expression patterns throughout these diverse areas. An unexpected singularity that also emerged from the Puelles et al. (2012b) analysis is that the *ventral premamillary nucleus*, which in the adult appears within the TuI subdomain, intercalated between the dorsal premamillary nucleus and the ventromedial nucleus, originates from the RM area within PHy, from where its cell population migrates tangentially *en masse* into the definitive THy locus.

At first glance it may seem that the complex molecular and fate regionalization of the hypothalamic basal plate is out of the ordinary, but recent detailed genoarchitectural studies of dorsoventral patterning in the basal spinal cord have similarly disclosed a diversity of molecularly distinct dorsoventral progenitor domains (actually also 5 in number), where characteristic cell types are produced (Ulloa and Briscoe, 2007; Dessaud et al., 2010; Grossmann et al., 2010). Similar studies of the hindbrain and midbrain basal plate likewise detect diverse dorsoventrally disposed progenitor domains or microzones (e.g., Sieber et al., 2007; Gray, 2008; Storm et al., 2009; Puelles et al., 2012a; Puelles, 2013). Such results probably also can be extrapolated to the diencephalic tegmentum [e.g., the dopaminergic cell populations are continuously produced along a mesodiencephalic tegmental continuum, which also produces various other cell populations, such as neurons associated to the fasciculus longitudinalis medialis, Nkx6.1/6.2-positive elements of the pre-Edinger–Westphal nucleus (Puelles et al., 2012a), cells associated to the red nucleus and to the medial terminal nucleus of the accessory optic tract (Puelles, 2013)]. Such heterogeneity hardly would result from a homogeneous basal progenitor population. Therefore, the complexity we see at the hypothalamic basal plate may be just a differentially developed (expanded) version of the general case along the whole neural tube. There surely is a differential role of the patterning effects exerted here by the prechordal plate (and the adenohypophysis afterwards) in explaining any properties that selectively apply to the basal hypothalamus. Further study is needed to investigate whether the five longitudinal basal subzones presently postulated in the hypothalamus can be extrapolated individually backwards into thinner corresponding domains in the other brain areas, examining as well how far the respective genoarchitecture is shared throughout (vs. regional differences). Since the hypothalamic basal plate is the largest dorsoventrally, it may well occur that ventralizing signals diffusing from the floor plate dorsalward, and secondary antagonistic interactions between transient early gene patterns (such as those observed in the spinal cord), can be read out by the responding basal matrix cells into more distinct levels of genomically significant signal concentrations. This would imply that smaller basal plates might have not only thinner, but perhaps also less longitudinal microzones. This issue will no doubt be cleared in the near future.

A final issue that should be commented in this section is the proposal of Kuhlenbeck (1973) that the tuberal/retrotuberal region is alar in nature, being separated from the mamillary/retromamillary region *sensu lato* by an alternative alar-basal boundary. This conclusion was also reached recently by Diez-Roux et al. (2011), due to the expression within the tuberal region of a number of genes otherwise characteristic of the alar plate, such as *Dlx* and *Arx* genes. This hypothesis certainly simplifies the concept of the hypothalamic basal plate, reducing it to the M/RM and PM/PRM longitudinal domains, but complicates instead the schema of the alar plate, which would then have five longitudinal zones (Pa, SPa and the three Tu/RTu subdomains). This hypothesis implies a lack of linearity (a step) in the alar-basal boundary at the preincertal/incertal hypothalamo-prethalamic border, an issue that will need additional analysis. The shared “alar” gene patterns in the tuberal region appear associated topographically to the sites where GABAergic neurons are produced (Puelles et al., 2012b). While no definitive explanation seems presently available for the fact that genes otherwise characteristic of the alar plate, such as *Arx* and *Dlx*, are expressed as well (with some differential characteristics) in the *Shh*-positive tuberal/retrotuberal territory, it is by no means extraordinary that, due to differential enhancer effects, the same gene can be activated independently in morphologically unrelated domains (for instance, *Shh* itself, held to be a ventral marker—even a floor plate marker by some authors—, is expressed also separately in the alar preoptic area). Such ectopic peculiarities should not confuse morphologic analysis. We are forced to take into account a variety of arguments in order to reach the most meaningful interpretations. Any single gene signal does not have a straightforward morphologic meaning. In this case, we hold as significant that there is a precocious molecular alar-basal division detectable in the forebrain already at neural plate stages, according to the early floor and basal expression of *Shh* plus a limiting band of *Nkx2.2* expression (Shimamura et al., 1995). This creates a primary pattern that was recently corroborated by progeny analysis of *Shh*- and *Foxb1*-derived populations (Zhao et al., 2008; Szabo et al., 2009), as well as chicken fate-mapping data (Sanchez-Arrones et al., 2009). These results pinpoint the dorsal limit of hypothalamic *Shh* expression, with the overlapping band of *Nkx2.2* expression (Figures 7A, 10B), as the primary alar-basal boundary. The added basal expression of *Arx*, *Dlx* and other gene markers listed by Diez-Roux et al. (2011) appears relatively later in development (after E10.5). We submit that this phenomenon may relate to differentiative decisions leading to the GABAergic phenotype adopted by many basal cells, mainly along the TuI/RTuI domain (the histaminergic neurons produced at the TuV/RTuV also share analogous markers).

### The *acroterminal hypothalamic domain* as a necessary causal background for the unique rostromedian hypothalamic specializations

**Figure 13:**
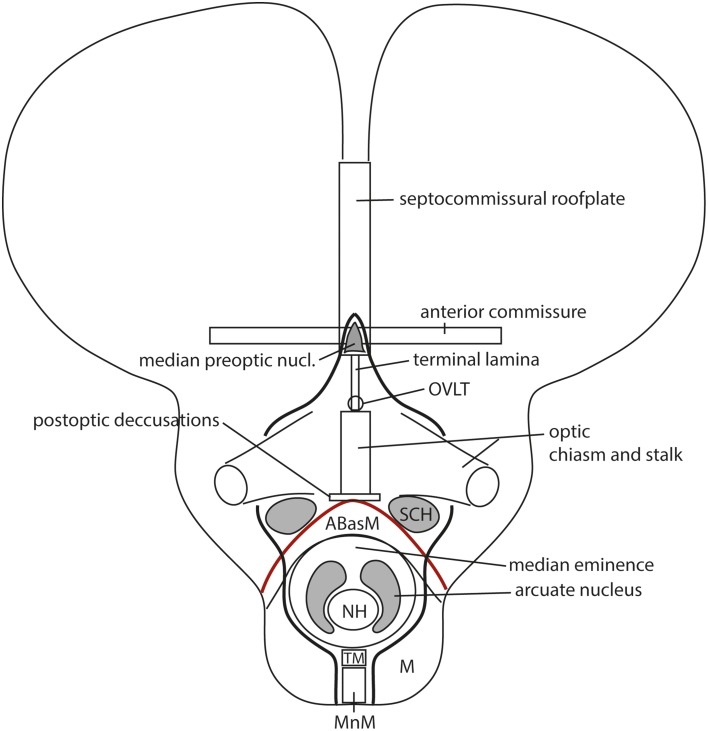
**Frontal schematic representation of the structures presently ascribed to the acroterminal domain (ATD); the latter is delimited right and left by a thick black line**. The alar-basal boundary is marked in red. The ATD starts at the preoptic roof, encompassing the anterior commissure bed and the median preoptic nucleus (MnPO); further down there is the terminal lamina, and probably also some other neighboring preoptic derivatives, ending with the organum vasculosum laminae terminalis (OVLT), a circumventricular specialization. The alar hypothalamic part of the ATD also includes the optic elements (eyes, stalks and chiasm) plus the postoptic decussations, and the suprachiasmatic nuclei (SCH) bilaterally. The basal ATD region includes the precociously differentiating median anterobasal area (ABasM), the median eminence, infundibulum, neurohypophysis (NH) and arcuate nuclei, plus the median tuberomamillary area (TM), finishing with the median mamillary area (MnM).

As mentioned above, the rostromedian hypothalamic midline stretching between the mamillary region (end of floor plate) and the anterior commissure (end of roof plate)—see Figure 9A—is singular in being patterned *dorsoventrally* (as opposed to anteroposteriorly, as is dictated by the columnar model—Figure 9B). Though neuroanatomic literature traditionally interprets this territory as extended along the length axis, due to the assumptions of the columnar model, its molecular patterning, which is already visible at neural plate stages (Puelles, 1995; Shimamura et al., 1995; Sanchez-Arrones et al., 2009) indicates instead that it should be understood as a singular rostromedian continuity of the lateral walls of the neural tube, representing the unpaired median place where the lateral walls—alar+basal—primarily meet each other rostrally, on top of the rostralmost floor plate and under the rostralmost roof plate (Figure 9A). This peculiar rostromedian domain belonging to the THy shows in the adult various structural specializations (Figure 13). In its alar subregion there is dorsally the *terminal lamina* and the *median preoptic nucleus* (TL; MnPO), as well as the *optic chiasm* (OCH), ventrally; the terminal lamina is fixed dorsally to the anterior commissure (roof plate) and ventrally to the optic chiasm. At the latter transitional neighborhood, the terminal lamina shows an intensely vascularized median circumventricular organ (the *organum vasculosum laminae terminalis;* OVLT). The ventral aspect of the optic chiasm relates intimately to the *postoptic decussations* (these are topologically rather “suboptic,” though they used to be named “supraoptic” in reference to the columnar axis); they apparently lie just above the alar-basal boundary (this is merely a tentative interpretation at this point, pending more detailed genoarchitectural analysis).

In its turn, the terminal median basal plate also shows a sequence of specializations: there is dorsally (close to the postoptic decussations) a median portion of the *anterobasal area* (ABasM; this is the primitive rostral end of the precociously differentiated hypothalamic cell cord, which used to be known as the “*retrochiasmatic area*,” e.g., in Puelles et al., 1987a; note ABas is a prosomeric-consistent term, though it was introduced by Altman and Bayer, 1986, whereas RCH is columnar). The ABas is horseshoe-shaped and displays bilateral wings within the TuD area of THy (Figure 10B). More ventrally, coinciding with the median part of TuI, there appears the *median eminence* and the associated *arcuate nucleus* (ME, Arc), as well as the *infundibulum* and the *neurohypophysis* (NH), whereas the underlying median TuV is represented by the tuberomamillary recess area (TM; Figure 13). Part of the *median mamillary region* (MnM) possibly participates of this extensive rostromedian (transverse) territory, immediately dorsal to the mamillary floor plate. Additional median or paramedian structures close to those described above also may be ascribed to the structurally singular rostromedian territory: for instance, the *optic vesicles* and their *stalks* (optic nerves), and the *suprachiasmatic nucleus* (SCH; Figure 13). Our criterion for adding the SCH to this singular region is that it is limited to a rostromedial sector of the THy, and does not reach the intrahypothalamic border. Accordingly, it needs a special causal underpinning, which most probably relates to the differential molecular profile of the rostromedian terminal area (see Ferran et al., 2015).

Indeed, these specializations in principle belong all to the THy, but they occupy a radially distinct territory at its rostralmost end, and none of them extend caudalwards across the whole THy, reaching the intrahypothalamic border. Their development must obey specific causes restricted to the rostromedian alar and basal midline and its immediate paramedian neighborhood. The differential histogenetic patterns observable at the standard THy entities that do reach the intrahypothalamic boundary (see list above) vs. the corresponding rostromedian specializations at each dorsoventral level are corroborated by the existence of developmental gene expression patterns distinguishing these two THy subregions (see Ferran et al., 2015). This might be construed eventually as evidence that the rostromedian hypothalamic terminal subdomain represents an extra, atypic hypothalamic neuromere (hp3). Though granting this possibility, we caution that finding support for this hypothesis would require to re-examine again the roof and floor plates, in order to verify that the requirement for a “complete” interneuromeric border can be satisfied (Puelles and Rubenstein, 2003). We have not obtained such evidence yet, so we keep this territory within hp2 and THy.

Meanwhile, it was thought convenient to have a specific name for this territory within the ampler concept of the terminal hypothalamic wall. Puelles et al. (2012b) proposed the novel term “*acroterminal hypothalamic domain*” (ATD), referring to its topologic location at the tip (Greek, *acron*) of the *terminal* wall. Accordingly, the descriptor “acroterminal” can be applied unambiguously to any of the mentioned specialized structures of this territory, as well as to the whole subregion, eschewing the continuous use of circumlocutions. Note the ATD is shared by the hypothalamus and the preoptic telencephalon (Figures 9A, 13). It is well possible that the ATD is a direct consequence of the signaling activity of the prechordal plate along the median part of the terminal wall.

**Figure 14:**
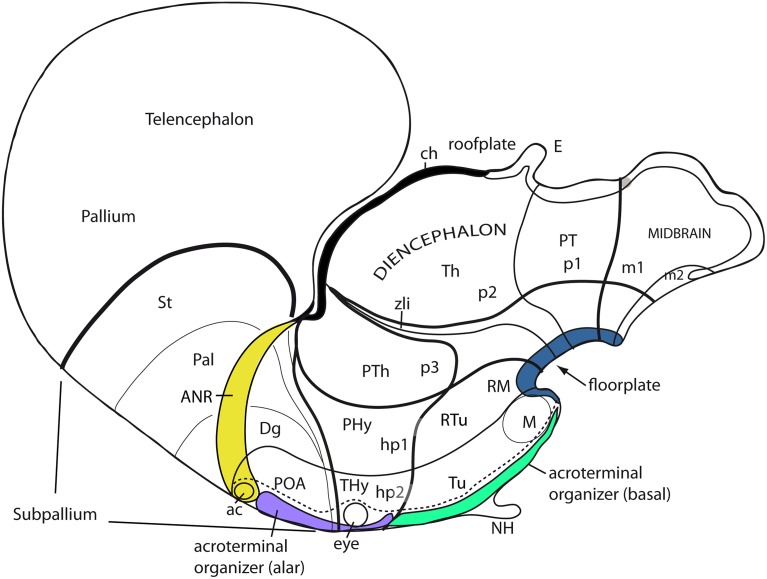
**Schema illustrating the apparent sources of patterning diffusible morphogens that may have effects on the hypothalamus**. The anterior neural ridge (ANR; yellow), which releases FGF8 is in fact a part of the roof plate (*dorsalizing influence*), rather than a source of AP effects; in contrast, the retromamillary and mamillary floor plate (dark blue associated to RM and M) releases SHH (*ventralizing influence*; note Shh secondarily also is expressed throughout the basal plate, and is later downregulated at the Tu area). We can speak of the acroterminal midline as a source of *AP patterning effects*. Recent observations (Ferran et al., 2015) show *Fgf18* expression within the postulated alar acroterminal organizer (fuchsia-labeled) and *Fgf8* and *Fgf10* expression within the postulated basal acroterminal organizer (green-labeled). There also are bilateral spots of *Fgf8* expression at the optic stalks (not shown).

Interestingly, both alar and basal parts of the ATD seem to develop signaling properties, due to the localized expression of several members of the fibroblast growth factor family (*Fgf8*, *Fgf10*, *Fgf18*; see Ferran et al., 2015; Figure 14). Diffusion of these morphogens caudalwards from the ATD into the hypothalamus may be relevant for its segmentation into hp1 and hp2, and/or for detailed anteroposterior patterning of the alar and basal hypothalamic territories. For instance, the difference between the median eminence/arcuate nucleus complex and the terminal dorsomedial domain might obey to FGF signaling from the local basal ATD area. The alar part of the ATD shows bilateral spots of *Fgf8* expression at the base of the optic stalks and a median line of *Fgf18* expression along the terminal lamina (see Ferran et al., 2015). Signaling spreading from these alar loci might be relevant for the differential specification of the median OVLT and the bilateral SCH nuclei.

## Coda

Looking into the rationale of the novel aspects in the prosomeric model possibly has brought us to consider quite unexpected morphological and developmental results, which seem relevant one way or other for underpinning solidly our assumptions about forebrain structure, including that of the hypothalamus, in a realistic causal background. Progress apparently lies in increasing our awareness of such relevant developmental phenomena and their spatial and molecular characteristics, incorporating them coherently after due analysis into the model's assumptions and predictions. This surely improves its overall consistency and sturdiness, to the advantage of potential morphologic interpretations and causal explanations. Our take-home message is that a morphologic model helps us to think all the better, the deeper its roots extend into causal foundations.

A good model points out the apparent best options for our dealings with complex reality (either the planning of our research, or the analysis of results), but certainly does not represent a definitive Truth that stops us from considering heterodox novel ideas and possible changes to the model. Models must adapt to progress in knowledge, or will be superseded. In the past, neuroanatomic models first aimed to encompass gross aspects of adult brain structure as they appeared in dissections, and accordingly were very much man-made and wanting in precision. Then they incipiently started to consider aspects of dorsoventral and anteroposterior developmental pattern (columnar versus neuromeric models), but were hampered by the low resolutive power of the research methods available, and possibly also by misguided (premature) attention to functions. Finally, the progress of molecular biology, genomics and mechanistic developmental biology has brought in masses of new relevant data, leading us to the consequent need of models capable of encompassing causal mechanisms of structure in three dimensions. We can no longer accept that the brain longitudinal axis, or any other fundamental structural component, be defined arbitrarily (e.g., merely implied by the use of given descriptors), without express reference to known molecular aspects of developmental causation, irrespective whether we only have tentative solutions, or seemingly solid ones. This is the modern, promising way in which we look at the hypothalamus now, in the new molecular scenario.

Since we have not yet collected or analyzed all possible data, we must be ready to change our assumptions as the model evolves in response to new techniques, additional experimental results and more detailed thought. Importantly, the morphological model of the hypothalamus should not be conditioned by functional preconceptions, as happened with the columnar model. Our justified interest in brain functions should find its proper place in the experimental analysis of the biology of living brain structure. Morphological models are important primarily as instruments to understand developing (evolving) brain structure. They allow us to produce increasingly detailed maps where causal mechanisms, differentiation patterns, connective pathways, synaptic fields and even neuro-pharmacological properties can be correlatively inscribed, first bi-dimensionally, and later in 3 dimensions. This complex and as yet incompletely fulfilled endeavor eventually should allow us to conceive multi-dimensional representations, which might be relevant for functional analysis, even though brain functions *per se*, representing dynamic capabilities of distributed interactive neural networks relative to the body and the world, hardly can find a fixed *place* in a morphological brain model.

**Quaerendo invenitis** (by asking, you will find) [J. S. Bach]

### Conflict of interest statement

The authors declare that the research was conducted in the absence of any commercial or financial relationships that could be construed as a potential conflict of interest.
